# Excited-State *Cis* and *Trans* Pt(IV) Diamine Anticancer Complexes

**DOI:** 10.1021/acs.inorgchem.5c01882

**Published:** 2025-05-28

**Authors:** Huayun Shi, Jana Kasparkova, Fortuna Ponte, Hana Kostrhunova, Guy J. Clarkson, Emilia Sicilia, Viktor Brabec, Peter J. Sadler

**Affiliations:** † Department of Chemistry, 12466 University of Warwick, Coventry CV4 7AL, U.K.; ‡ State Key Laboratory of Vaccines for Infectious Diseases, Xiang An Biomedicine Laboratory, National Innovation Platform for Industry-Education Integration in Vaccine Research, School of Public Health, Xiamen University, Xiamen 361102, China; § Department of Biophysics, Faculty of Science, Palacky University, CZ-77900 Olomouc, Czech Republic; ∥ Institute of Biophysics, 86853 Czech Academy of Sciences, CZ-61200 Brno, Czech Republic; ⊥ Department of Chemistry and Chemical Technologies, University of Calabria, via Pietro Bucci, 87036 Arcavacata di Rende , Cs, Italy

## Abstract

Classical structure–activity relationships for square-planar Pt­(II) anticancer complexes were based on the activity of *cis*-[PtCl_2_(NH_3_)_2_] (cisplatin) and inactivity of the *trans* isomer. Many other families of *cis*-diamine complexes and analogous octahedral Pt­(IV) prodrugs are active. Here, we report the chemical and biological activities of isomeric photoactivatable *cis,trans,cis*- and all-*trans*-[Pt­(N_3_)_2_(OH)_2_(MNZ)_2_] complexes (MNZ = metronidazole, 1-(2-hydroxyethyl)-2-methyl-5-nitroimidazole). While both are relatively nontoxic in the ground state, only the all-*trans* isomer is cytotoxic toward bladder cancer cells on excitation with visible light and under hypoxia. Studies of DNA interstrand cross-links and photocytotoxicity toward wild-type and nucleotide-excision-repair deficient cells suggest that, unlike cisplatin, DNA is not the major target site of these isomers. Differences in photoactivation pathways were also explored using time-dependent DFT calculations. The key differences between the isomers on irradiation are the more rapid photoactivation of the all-*trans* complex, generation of azidyl radicals, retention of its metronidazole ligands, higher accumulation in cancer cells, binding to DNA, RNA, and proteins, and induction of apoptosis and mitochondrial membrane damages. These findings provide a basis for the design of future photochemotherapeutic platinum anticancer prodrugs.

## Graphical abstract

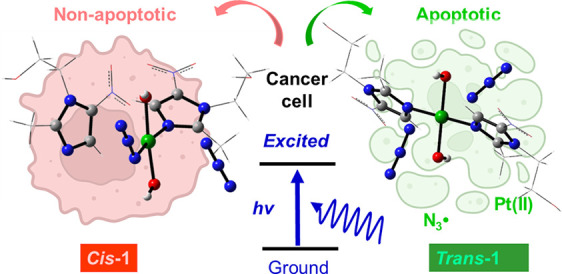

## Introduction

The activity of *cis* versus *trans* isomers has long attracted attention in the design of platinum diamine anticancer drugs.^1–3^ The first FDA-approved drug cisplatin, *cis*-[PtCl_2_(NH_3_)_2_] has *cis* ammine ligands.^4–6^ In contrast, its *trans* isomer, transplatin, is inactive.^5–7^ The inability of the *trans* complex to form lethal intrastrand GG DNA cross-links is due to the difference in location of the reactive chlorido ligands, and more rapid repair of transplatin lesions.^2,8^ In early structure–activity relationship studies, the *cis* diamine geometry appeared to be essential for anticancer activity. Cisplatin undergoes hydrolysis to generate more reactive aqua and diaqua complexes which form especially intrastrand GG cross-links, and unwind and bend DNA. High mobility group (HMG) proteins then bind strongly in the hydrophobic DNA platination site by phenylalanine insertion, leading to apoptotic cell death.^9^

It is now well established that certain amine ligands can promote the activity of *trans*-Pt­(II) complexes, including heteroaromatic *N*-donors, iminoether ligands, and asymmetric aliphatic amine ligands.^1,2,10–16^ Their different mechanisms of action allow them to circumvent cisplatin resistance. Interestingly, UVA irradiation promotes the loss of the second chloride from transplatin and increases the formation of interstrand DNA cross-links, ultimately leading to cytotoxicity comparable with cisplatin.^8^ Kinetically inert Pt­(IV) complexes can undergo reduction to form cytotoxic Pt­(II) species and display reduced side effects and improved cancer selectivity.^17–21^ Early studies on the isomeric pair of Pt­(IV) prodrugs [Pt^IV^Cl_2_(OH)_2_(cyclohexylamine)­(NH_3_)] (all-*trans*, **JM335** and *cis,trans,cis*, **JM149**) showed that the all-*trans* isomer **JM335** is more cytotoxic and relatively less cross-resistant against both acquired and intrinsic cisplatin-resistant cells compared to the *cis* isomer.^11,22^ **JM335** would be reduced rapidly in cells and form reactive hydroxido/aqua adducts which then bind to DNA.^23^ In contrast, the less potent anticancer complex *cis,trans,cis*-isomer **JM149** does not undergo observable reduction under similar conditions.

Diodido diamine Pt­(IV) complexes, such as *trans,cis*-[Pt­(OH)_2_I_2_(en)], undergo reduction on irradiation with visible light, but are also readily reduced by thiols such as GSH.^24–26^ In contrast, diazido Pt­(IV) complexes exhibit high dark stability in the presence of GSH and are photocytotoxic upon irradiation, generating azidyl radicals and toxic Pt­(II) species.^27–30^ All-*trans* photoactive diazido dihydroxido diamine Pt­(IV) complexes appear to have higher aqueous solubility, a more intense and red-shifted LMCT band, and higher photocytotoxicity compared with their *cis* isomers.^31,32^ The substituents on the *N*-heterocyclic amine ligands modify the photocytotoxicity of diazido Pt­(IV) complexes significantly, and we found previously that a complex with *trans* nitroimidazole ligands exhibited low dark cytotoxicity and promising photocytotoxicity under both normoxia and hypoxia.^33^

We have synthesized novel photoactivatable Pt­(IV) diazido diamine dihydroxo complexes with nitroimidazole ligands, all-*trans*-[Pt­(N_3_)_2_(OH)_2_(MNZ)_2_] (*Trans*-**1**) and *cis*,*trans*,*cis*-[Pt­(N_3_)_2_(OH)_2_(MNZ)_2_] (*Cis*-**1**, Figure 1a). Metronidazole (1-(2-hydroxyethyl)-2-methyl-5-nitroimidazole, MNZ) is a clinical antimicrobial prodrug which undergoes activation by nitro-group reduction under hypoxia in vivo.^34^ The Pt­(II) complex *trans*-[PtCl_2_(MNZ)_2_] exhibits enhanced activity compared to metronidazole itself against the anaerobic parasite E. histolytica which causes amoebiasis.^35^ Complexation of metronidazole to Pt­(II) in *cis*-[PtCl_2_(NH_3_)­(MNZ)] enhances targeting to DNA, cytotoxicity in hypoxia and radiosensitization activity.^36,37^ An axial metronidazole conjugated to a Pt­(IV) prodrug can be irreversibly reduced under hypoxic conditions to retain the Pt­(IV) prodrug in areas of hypoxia, and accumulate deep within cancer cell spheroids.^38^ Photoactive Pt­(IV) prodrugs containing a bound metronidazole ligand have not been reported.

**Figure 1:**
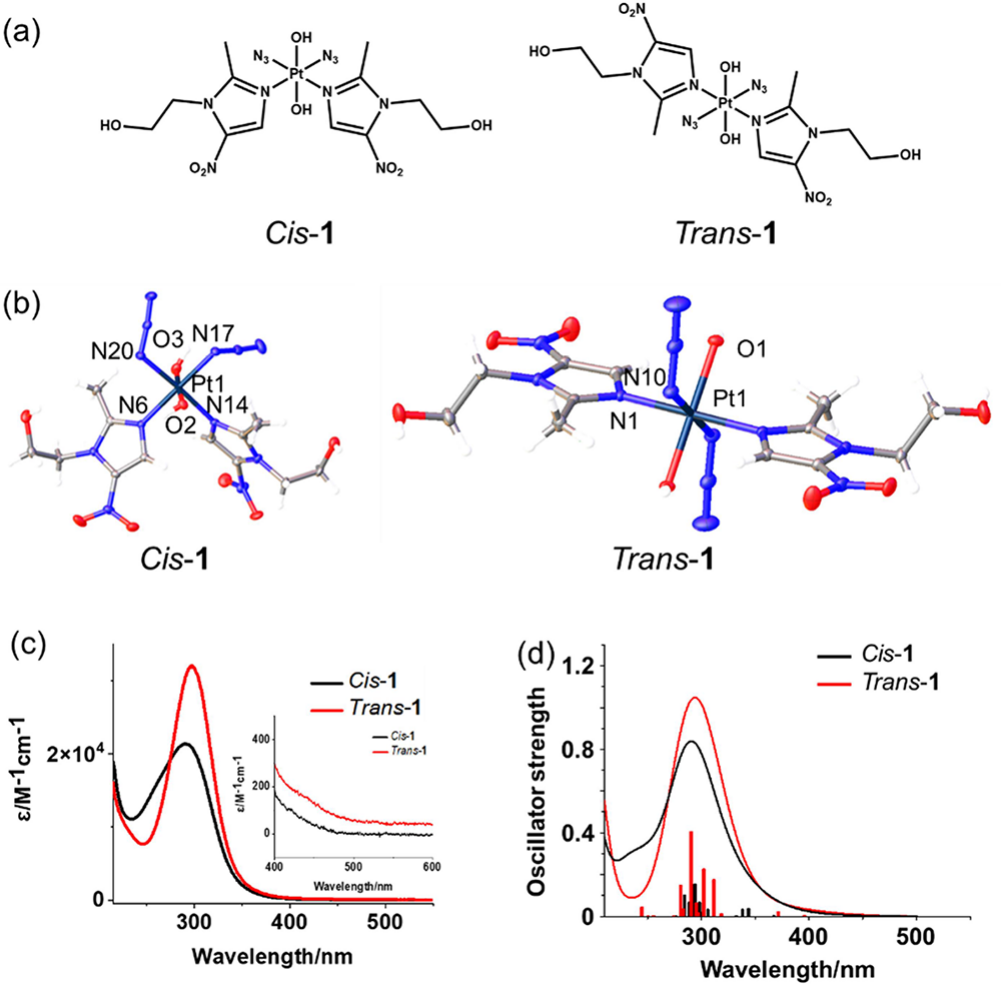
(a) Chemical structures, (b) X-ray crystal structures of *Cis*-**1** and *Trans*-**1**·2MeOH (with with thermal ellipsoids drawn at 50% probability and MeOH not shown), (c) observed UV–vis spectra for the photoactive *cis* and *trans* diazido Pt­(IV) isomers in water, and expansion of the 400–500 nm region (insert), and (d) calculated UV–vis spectra at the B3LYP/6–31G* (SDD for Pt) level of theory.

Here, we compare the contrasting chemical and biological properties of the *cis* and *trans* dimetronidazole isomers, *Cis*-**1** and *Trans*-**1**, using experimental and computational methods, to elucidate the factors which contribute to the high photocytotoxic anticancer activity of the all-*trans* isomer. These include differences in structure, lipophilicity, redox potentials, ROS and radical generation, electronic transitions including Time-dependent Density Functional Theory (TDDFT) calculations, cell accumulation, DNA binding, and effect on mitochondrial membrane potentials, with studies in the dark and upon photoirradiation in normoxia and hypoxia.

## Results

### Synthesis and Characterization

*Cis* and *trans* isomers were prepared via the synthetic routes in Scheme S1. The synthesis of *Cis*-**1** began with K_2_PtCl_4_ and 2 mol. equiv of metronidazole, while that of *Trans*-**1** began with K_2_PtCl_4_ and 8 mol. equiv of metronidazole, followed by treatment with HCl to ensure a resulting *trans* configuration. Both complexes had satisfactory HPLC purity (>99%, Figure S1) and were fully characterized by ESI-HR-MS (Figure S2), ^1^H and ^13^C NMR (Figures S3–6), X-ray crystallography (Figure 1b, Tables S1–3), and UV–vis spectroscopy (Figure 1c).

*Trans*-**1** and *Cis*-**1** have similar HPLC retention times, suggesting similar lipophilicities. Their NMR peak patterns are similar, but the chemical shifts are affected by the configuration. Generally, ^1^H NMR signals for *Trans*-**1** are shifted to lower field compared with those for *Cis*-**1**. The proton on the Pt-coordinated imidazole ring is a ^1^H NMR singlet with ^195^Pt satellites, at 8.16 ppm for *Cis*-**1** and 8.31 ppm for *Trans*-**1**, and the *J* ^195^Pt–^1^H coupling constant for this peak increases from 15.3 to 17.8 Hz when the configuration changes from *cis* to *trans*. An apparent difference in the ^1^H NMR signals of the methyl group in the metronidazole ligands was induced by the configurational change (*Cis*-**1**: 2.58 ppm; *Trans*-**1**: 3.04 ppm). Crystals suitable for X-ray diffraction studies of complexes *Cis*-**1** and *Trans*-**1**·2MeOH were obtained through evaporation of the corresponding solutions in methanol. The Pt atom sits on an inversion center for *Trans*-**1** with azido, hydroxido and metronidazole ligands in symmetric *trans* positions. In contrast, *Cis*-**1** is nonsymmetric with *cis* azido and *cis* metronidazole ligands. The corresponding bond lengths are similar in the isomers. The two metronidazole ligands form 53.2 and 47.1° angles with the equatorial plane due to steric effects, whereas in *Trans*-**1** the rings lie in the equatorial plane (Figure 1b). Hydrogen bonds are formed between the OH of methanol and Pt–OH or metronidazole OH in *Trans*-**1**·2MeOH, while for *Cis*-**1** hydrogen bonds are formed between Pt–OH and metronidazole OH (Table S3).

Based on the X-ray crystal structures, the optimized ground state structures (Figure S7) of both isomers were obtained by DFT calculations using the selected hybrid B3LYP functional. Their experimental and simulated electronic absorption spectra (Figure 1d, Tables S4 and S5), are in very good agreement (Figure 1c,d). An apparent red-shift and increase of UV–vis absorption maximum band in H_2_O is observed when the configuration changes from *cis* (290 nm, ε = 21,389 M^–1^ cm^–1^) to *trans* (297 nm, ε = 31,818 M^–1^ cm^–1^). Notably, the absorbance at 420, 465, and 520 nm for these isomers is very weak, but not zero, except for *Cis*-**1** at 520 nm (*Cis*-**1/** *Trans*-**1**: 420 nm (96/190 M^–1^ cm^–1^); 465 nm (17/96 M^–1^ cm^–1^); 520 nm (0/54 M^–1^ cm^–1^)).

For *Cis*-**1**, the maximum absorption wavelength (λ_max_) is observed at 456 nm (Tr1) with a low corresponding oscillator strength of only 0.001, indicating a relatively weak transition. This excitation corresponds to an electronic transition described as HOMO­(H) → LUMO + 2 (L + 2), with a calculated contribution of 96%. The band centered at 286 nm, however, is a mixed band, involving transitions with significant ligand-centered (LC) and ligand-to-ligand charge transfer (LLCT) character. An analysis of the Natural Transition Orbitals (NTOs)^39^ reveals that for the LC excitations (Table S6), only the metronidazole ligand is involved in the transitions. While for the LLCT transitions, the electron density is predominantly transferred from the OH/N_3_ groups to the metronidazole ligands. Absorption bands at 456 nm (Tr1), 298 nm (Tr6), 289 nm (Tr9) and 255 nm (Tr12) contain transitions with ligand-to-metal charge transfer (LMCT).

The λ_max_ of *Trans*-**1** is 408 nm with an oscillator strength of 0.006 (Tr1), this electronic transition primarily involves the HOMO­(H) → LUMO + 1 (L + 1) with a contribution of 98%. The absorption band centered at 291 nm is attributed to spin-allowed mixed LLCT and LC excitations, but it is not possible to assign a definitive character to this band, as all the platinum-associated ligands participate in the transitions (Table S7). The N_3_-to-Pt LMCT transitions are found at absorption wavelengths of 295 nm (Tr5) and 281 nm (Tr7), while the MNZ to Pt LMCT is at 302 nm (Tr4). The molecular orbitals associated with the metronidazole ligand play a more crucial role in *Cis*-**1**, compared with its *trans* isomer.

### Redox Potentials, Dark Stability, and Photoactivation

The cyclic voltammograms of *Cis*-**1**, *Trans*-**1** and metronidazole (1 mM) in N_2_-saturated DMF were acquired at 298 K, using 0.1 M NBu_4_PF_6_ as supporting electrolyte (Figure S8, Table S8). Metronidazole shows a reversible wave due to the reduction of the nitro group, assignable to Ar-NO_2_/Ar-NO_2_^·–^, with *E*_pa_: −1.52 V; *E*_pc_: −1.61 V. Similar reversible reduction waves ascribed to the nitro group were observed for both *Cis*-**1** (*E*_pa_: −1.45 V; *E*_pc_: −1.60 V) and *Trans*-**1** (*E*_pa_: −1.42 V; *E*_pc_: −1.61 V), while irreversible reduction waves assigned to Pt^IV^/ Pt^II^ were observed for *Cis*-**1** and *Trans*-**1** at −1.27 and −1.28 V, respectively.

Both *Cis*-**1** and *Trans*-**1** exhibit high dark stability in both air- and nitrogen-saturated aqueous solutions at 310 K as monitored by UV–vis spectroscopy for 2 h (Figure S9) and by LC-MS for 24 h (Figure S10). In addition, high dark stability in the presence of 2 mM (40× molar excess) GSH was observed for both complexes (Figure S11). Upon irradiation with blue light (463 nm), there was an apparent decrease in intensity of the LMCT band for both complexes, indicating the release of azido ligands from Pt and the reduction of Pt­(IV). Photoactivation of *Trans*-**1** was faster than for *Cis*-**1**, with the decrease in absorbance reaching a plateau within 40 min, while reaching a plateau for *Cis*-**1** took 90 min (Figure 2a–c). With green light (517 nm), the LMCT band of *Trans*-**1** decreased by 16% after 120 min irradiation, while that of *Cis*-**1** decreased by only 5% (Figure 2d–f). No apparent difference was observed between the photoactivation of air- and nitrogen-saturated solutions with blue light, while a faster rate was observed for *Trans*-**1** (Figure S12) with green light irradiation. Only two main photoproducts were detected by LC-MS for *Cis*-**1**, [Pt^II^(MNZ)­(HCOOH)_2_(HO)]^+^ (peak c1 in Figure S12, *m*/*z* 474.93) and {MNZ + H^+^} (c2, *m*/*z* 171.92)), while five photoproducts [Pt^III^(MNZ)_2_(HCOO)_2_]^+^ (t1, *m*/*z* 627.05); [Pt^III^(MNZ)_2_(OH)_2_]^+^ (t2, *m*/*z* 571.05); [Pt^III^(MNZ)_2_(HCOO)­(N_3_)]^+^ (t3, *m*/*z* 624.09); [Pt^II^(MNZ)_2_(CH_3_CN)­(N_3_)]^+^ (t4, *m*/*z* 620.10); and {[Pt^II^(MNZ)_2_(OH)_2_] + Na}^+^ (t5, *m*/*z* 594.12) were detected for *Trans*-**1** (Figure S13, Tables S9 and S10, HCOOH and CH_3_CN are from HPLC eluants). Notably, released metronidazole was observed only for *Cis*-**1**, while in all Pt photoproducts from *Trans*-**1**, the two metronidazole ligands are retained.

**Figure 2:**
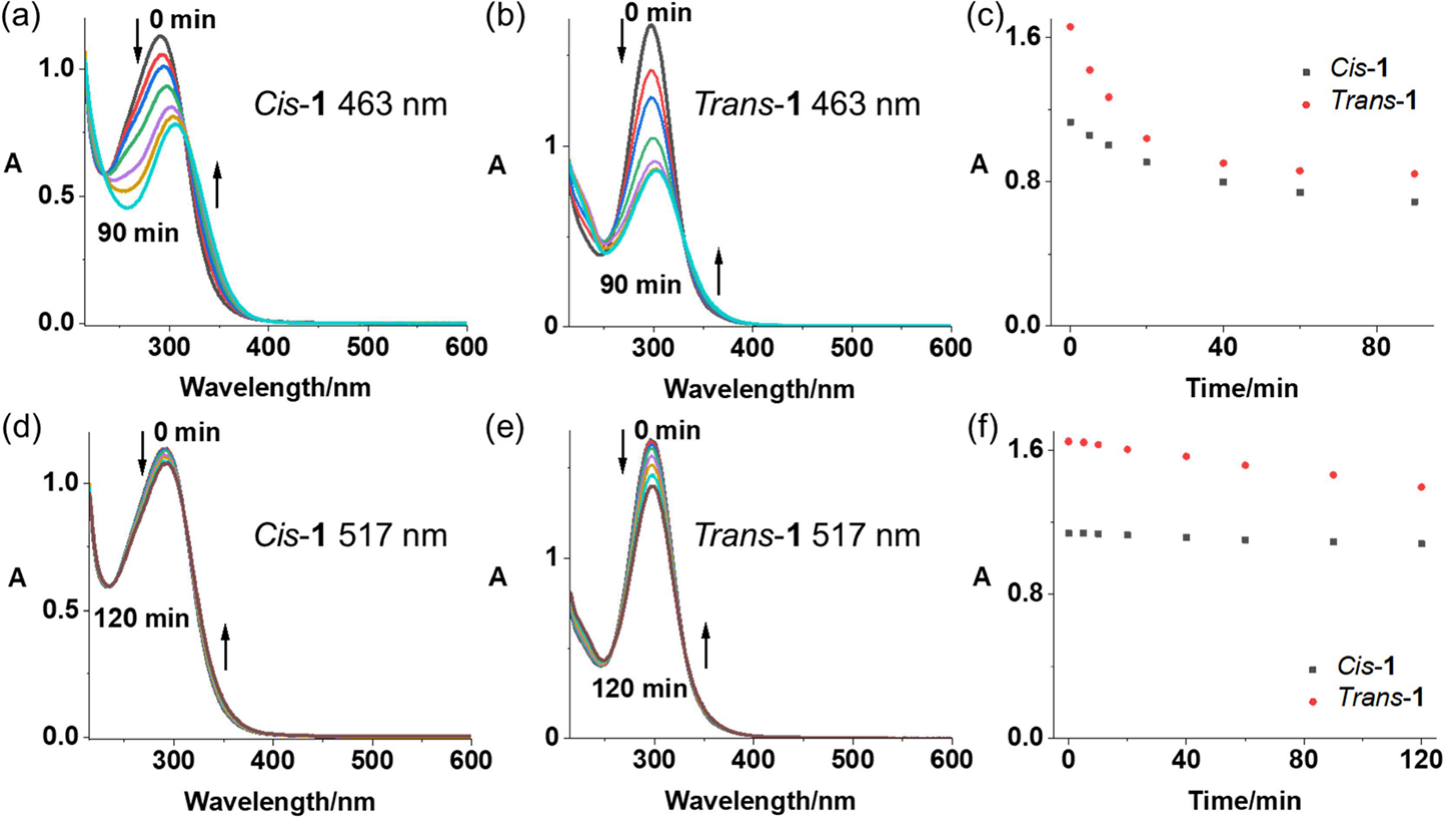
Time-dependent UV–vis spectral changes for complexes *Cis*-**1** and *Trans*-**1** in air-saturated MillQ H_2_O exposed to blue light (463 nm): (a) *Cis*-**1**, (b) *Trans*-**1** or green light (517 nm), (d) *Cis*-**1**, (e) *Trans*-**1** at 298 K. (c) and (f) Plots of time-dependent absorbance changes for complexes at the absorbance maximum (290 nm for *Cis*-**1**; 297 nm for *Trans*-**1**) upon irradiation with light of different wavelengths ((c) 463 nm; (f) 520 nm).

Azidyl radicals play an important role in the mechanism of action of photoactivated diazido Pt­(IV) complexes.^40^ Attempts were made to detect azidyl radicals generated during photoactivation of complexes *Cis*-**1** and *Trans*-**1** in aqueous solution by trapping with DMPO and detecting by EPR (Figure 3). With continuous blue light (463 nm) irradiation, azidyl radicals were detected for *Trans*-**1** as DMPO-N_3_·radicals which give a 1:2:2:1 quartet of triplets. In contrast, no DMPO-N_3_^·^ radicals were observed for *Cis*-**1**.

**Figure 3:**
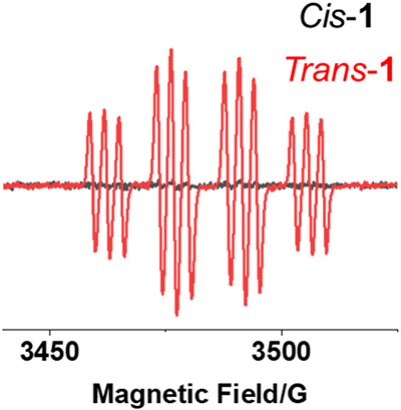
EPR spectra of complexes *Cis*-**1** or *Trans*-**1** (2.5 mM) in the presence of DMPO (40 mM) in Milli-Q water (containing 5% DMSO) showing the formation of DMPO–N_3_^·^ adducts for *Trans*-**1** (red), but not *Cis*-**1** (black), after irradiation (463 nm, 10 min).

### Excited States and Photoactivation Mechanisms

Upon irradiation with visible light, the singlet excited states of the two isomers are populated (Table S11). The S1 state was identified as the bright state of *Cis*-**1**, whereas for *Trans*-**1** the S4 state is the bright one. Due to the presence of the platinum center, the singlet excited states should undergo very efficient intersystem crossing (ISC) onto the triplet manifold populating the triplet states (Table S12). The mixed character of each state was confirmed by the TheoDORE analysis, and is evident in the donor–acceptor NTO plots (Tables S13–16). The two triplet states, denoted as T1 and T2, that are closest in energy to the bright S1 state of *Cis*-**1**, were investigated using the vertical approximation. In contrast, seven triplet states were examined for *Trans*-**1**. To evaluate the feasibility of ISC processes from bright states to the triplet manifold, spin–orbit coupling (SOC) matrix elements and adiabatic energy differences between the singlet and triplet states were calculated (Table S17). The high values of the SOC matrix elements, induced by the presence of the platinum atom, facilitate the crossing and therefore the population of triplet states. After optimization using the TDDFT approximation and unrestricted Kohn–Sham (UKS) approach,^41,42^ two triplet states were obtained for *Cis*-**1**, labeled *Cis*-T_a_ and *Cis*-T_b_, while optimizations for *Trans*-**1** collapsed into a single triplet state, named *Trans-*T_a_ (Figure 4, Table S18).

**Figure 4:**
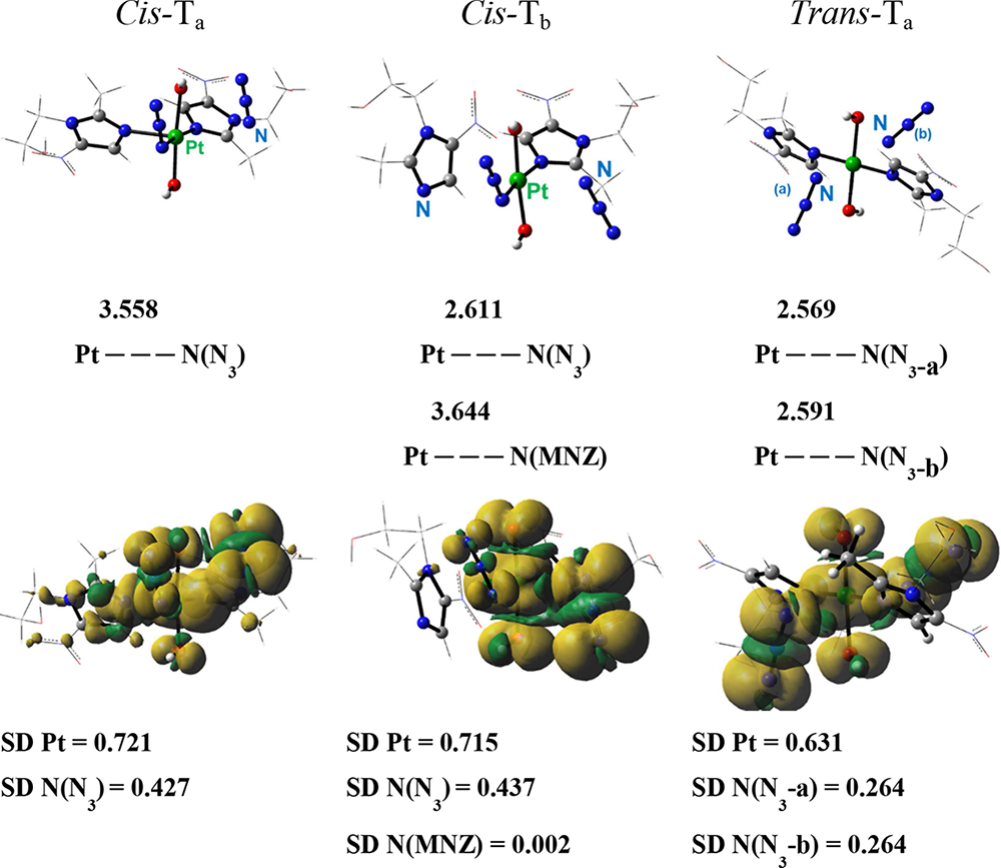
Structures of triplet states *Cis*-T_a_, *Cis*-T_b_, and *Trans*-T_a_, optimized within the unrestricted DFT formalism, together with the most important bond lengths (Å) (top) and computed spin density distributions of the intercepted triplet states together with the most important spin density values (SD in a.u.) on the platinum metal center and the atoms of the coordinated ligands (bottom).

All the observed triplet states exhibit a dissociative character with at least one ligand released from each structure. Upon irradiation, *Cis*-**1** can rapidly release one of the two azido ligands, forming the [Pt^III^(MNZ)_2_(OH)_2_(N_3_)] species and the azidyl radical. This rearrangement, derived from the triplet *Cis*-T_a_, is accompanied by an elongation of the Pt–N­(N_3_) bond (3.558 Å). The Mulliken spin density values clearly indicate the localization of one unpaired electron on Pt and another on the azido ligand. In the dissociative *Cis*-T_b_ triplet state, more pronounced geometric changes are observed. The elongation of the Pt–N­(N_3_) and Pt–N­(MNZ) bond lengths indicates that both azido and metronidazole ligands are photoreleased, leading to the formation of the reduced complex [Pt^II^(MNZ)­(OH)_2_N_3_]^−^. Mulliken spin density values reveal that the unpaired electrons are primarily localized on the Pt center (ca. 0.7 a.u.) and on the nitrogen atoms of the released azido ligand (ca. 0.4 a.u.). This triplet structure is in equilibrium with the adduct where the platinum center is fully reduced, and the azido and metronidazole ligands leave the metal coordination sphere as radicals.

In contrast, both azido ligands are released from *Trans-* **1** when irradiated to form the reduced complex *trans*-[Pt^II^(MNZ)_2_(OH)_2_]^+^. The Mulliken spin density of ca. 0.3 a.u. on the N atom of the azido ligands, together with the relative plot, show the unpaired electrons to be equally localized on the azido ligands. Potentially viable pathways leading to the formation of the Pt­(II) species detected by LC-MS are illustrated in Figure S14 based on the outcomes of DFT calculations. Figure S14 shows how Pt­(II) photoproducts formed by photoreduction of the two isomers in their triplet states might react with azidyl radicals to produce other Pt­(II) species.

### Photocytotoxicity, Cellular Accumulation and Distribution, and Cell Death Mechanism

The photocytotoxicity of *Cis*-**1** and *Trans*-**1** under normoxic (21% O_2_) and hypoxic (1% O_2_) environments toward human cancer cell lines, including human A2780 and cisplatin resistant A2780cis ovarian, and SW780 bladder cancer cells was investigated upon irradiation with blue light (465 nm, 4.8 mW cm^–2^) and green light (520 nm, 11.7 mW cm^–2^) in comparison with parent complex all-*trans*-[Pt­(N_3_)_2_(OH)_2_(pyridine)_2_] (**FM190**), and cisplatin (Tables 1 and S19). Low dark cytotoxicity with IC_50_ values >100 μM was observed for both isomers toward all cancer cell lines under different oxygen concentrations.

**Table 1:** IC_50_ Values and Photocytotoxicity Indices (PI) for Complexes in SW780 Bladder Cancer Cells after 1 h Incubation, 1 h Irradiation (Blue 465 nm, Green 520 nm) and 72 h Further Incubation under Normoxia (21% O_2_) and Hypoxia (1% O_2_)­^a^

			IC_50_ (μM)^b^			
condition			*Cis*-1	*Trans*-1	FM190	CDDP
normoxia (21% O_2_)	dark		>100	>100	>50	2.5 ± 0.4
	465 nm		>100	12.0 ± 1.2	7.4 ± 0.9	3.1 ± 0.1
	520 nm		>100	66.5 ± 1.4	46.3 ± 5.5	3.0 ± 0.3
	PI	blue		>8.3	>6.7	
		green		>1.5	>1.07	
hypoxia (1% O_2_)	dark		>100	>100	82.2 ± 4.8	12.1 ± 3.0
	465 nm		>100	12.7 ± 0.9	13.3 ± 2.6	14.2 ± 4.5
	520 nm		>100	>100	60.5 ± 0.5	13.5 ± 7.9
	PI	blue		>7.8	6.2	
		green			1.4	

a CDDP (cisplatin) and **FM190** were studied for comparison.

b All IC_50_ values were obtained from at least two independent experiments.

However, under visible light irradiation, only *Trans*-**1** exhibited photocytotoxicity toward SW780 bladder cancer cells similar to **FM190**, regardless of light wavelength and oxygen concentration. In contrast, *Cis*-**1** showed no apparent cytotoxicity upon irradiation under the same conditions. For ovarian cancer cells, moderate photocytotoxicity with an IC_50_ of ca. 45 μM was observed for *Cis*-**1** only with blue light exposure, about 2–5× less effective than for *Trans*-**1**. With green light irradiation, only *Trans*-**1** was cytotoxic toward A2780 cells, but *Cis*-**1** was not toxic. Notably, no apparent difference in photocytotoxicity of *Cis*-**1** in wild type A2780 and cisplatin-resistant A2780cis cells was seen, while *Trans*-**1** was ca. 4× less toxic toward A2780cis, perhaps suggesting different mechanisms of action.

The cellular accumulation of Pt from *Cis*-**1** and *Trans*-**1** (10 μM) was investigated for human SW780 bladder cancer cells under both normoxia and hypoxia (Table S20). After 2 h incubation in the dark, Pt accumulation in cells treated with *Trans*-**1** was ca. 3× and 2× higher than that for *Cis*-**1** under normoxia and hypoxia, respectively. Surprisingly, cellular Pt accumulation of these two complexes showed no apparent difference after irradiation (465 nm) in normoxia, while a ca. 2× enhancement was observed in hypoxia. The cellular distribution of *Cis*-**1** and *Trans*-**1** (10 μM) in SW780 cells in the dark was also investigated (Table S21). Interestingly, *Trans*-**1** displayed a relatively higher ratio of Pt accumulation in nuclei (49.5 ± 6.2%) in the dark than *Cis*-**1** (37.2 ± 4.3%).

The mitochondrial membrane potential is a key indicator of mitochondrial function, which affects the energy production of cells, and therefore cellular activity.^43^ Tetramethylrhodamine ethyl ester (TMRE) cations accumulate in negatively charged mitochondria.^44^ Upon Δψ depolarization, TMRE redistributes from mitochondria to cytosol, then the extracellular medium, and finally results in reduced cellular fluorescence. When SW780 cells were treated with *Cis*-**1** and *Trans*-**1** (20 μM) in the dark for 74 h under normoxia or hypoxia, no apparent difference from the negative control was observed (Figure S15). However, upon irradiation, a significant decrease in fluorescence intensity was observed for cells treated with *Trans*-**1** under normoxia, while the effect of irradiated *Cis*-**1** was much less. Compared with normoxia, a mitochondrial membrane potential change was also observed under hypoxia for cells treated with irradiated *Trans*-**1**, but the shift of the major fluorescence peak was not as significant as that under normoxia. A small portion of cells showed completely quenched signal, indicating an effect of oxygen on the mitochondrial membrane potential changes induced by *Trans*-**1** upon irradiation (Figure S16). In addition, no apparent fluorescence signal change was observed for cells treated with irradiated *Cis*-**1** under hypoxia. When using a lower concentration (10 μM) under normoxia, no mitochondrial membrane potential change was seen in the dark. Cells treated with *Trans*-**1** exhibited a decreased fluorescence intensity, but the change was less significant at the lower concentration. In contrast, cells treated with *Cis*-**1** and light alone showed no change in mitochondrial function, indicating low cytotoxicity.

Apoptosis was characterized using dual staining of Annexin V-FITC and PI in SW780 bladder cancer cells treated with *Cis*-**1** and *Trans*-**1** (20 μM) in the absence or presence of light (Figure S17). For cisplatin, Pt-DNA binding is regarded as the major trigger of apoptosis.^45^ Incubation with *Cis*-**1** or *Trans*-**1** in the dark (2 + 72 h) did not cause significant changes in the status of cells, which matches well with their low dark cytotoxicity. Upon irradiation for 1 h with blue light, the majority of cells treated with *Cis*-**1** remained healthy, while the population of cells at the late apoptosis stage increased from 16.9 to 61.0% and for dead cells increased from 3.7 to 12.4% after treatment with *Trans*-**1** and irradiation. In contrast, the population of cells remaining healthy decreased from 71.1 to 25.5%. Irradiation alone had no significant effect on the apoptosis stage of cells.

### DNA Binding

DNA is recognized as an important target for cisplatin and other classical platinum drugs, especially its guanine bases.^4,46,47^ First, binding of the mononucleotide guanosine monophosphate (5′-GMP) to the photoactivatable isomers was studied. Two Pt-GMP adducts, [Pt­(MNZ)_2_(GMP)­(HCOOH)­(N_3_)]^+^ (*m*/*z* 972.05, a) and [Pt­(MNZ)_2_(CH_3_CN)­(OH)­(GMP-H)]^+^ (*m*/*z* 942.12, b), were detected by LC-MS for reactions of *Trans*-**1** with 2 mol. equiv of 5′-GMP in aqueous solution, after 60 min blue light (463 nm) irradiation (Figure 5b). No Pt-GMP adducts were observed for *Cis*-**1** under the same conditions (Figure 5a). In a cell-free medium, a ca. 3× higher amount Pt bound to calf thymus DNA (ctDNA) when treated with *Trans*-**1** than for *Cis*-**1**, after incubation and irradiation with blue light (465 nm) for 1 h and further incubation in the dark, while no Pt binding was detected in the dark (Figure 5c).

**Figure 5:**
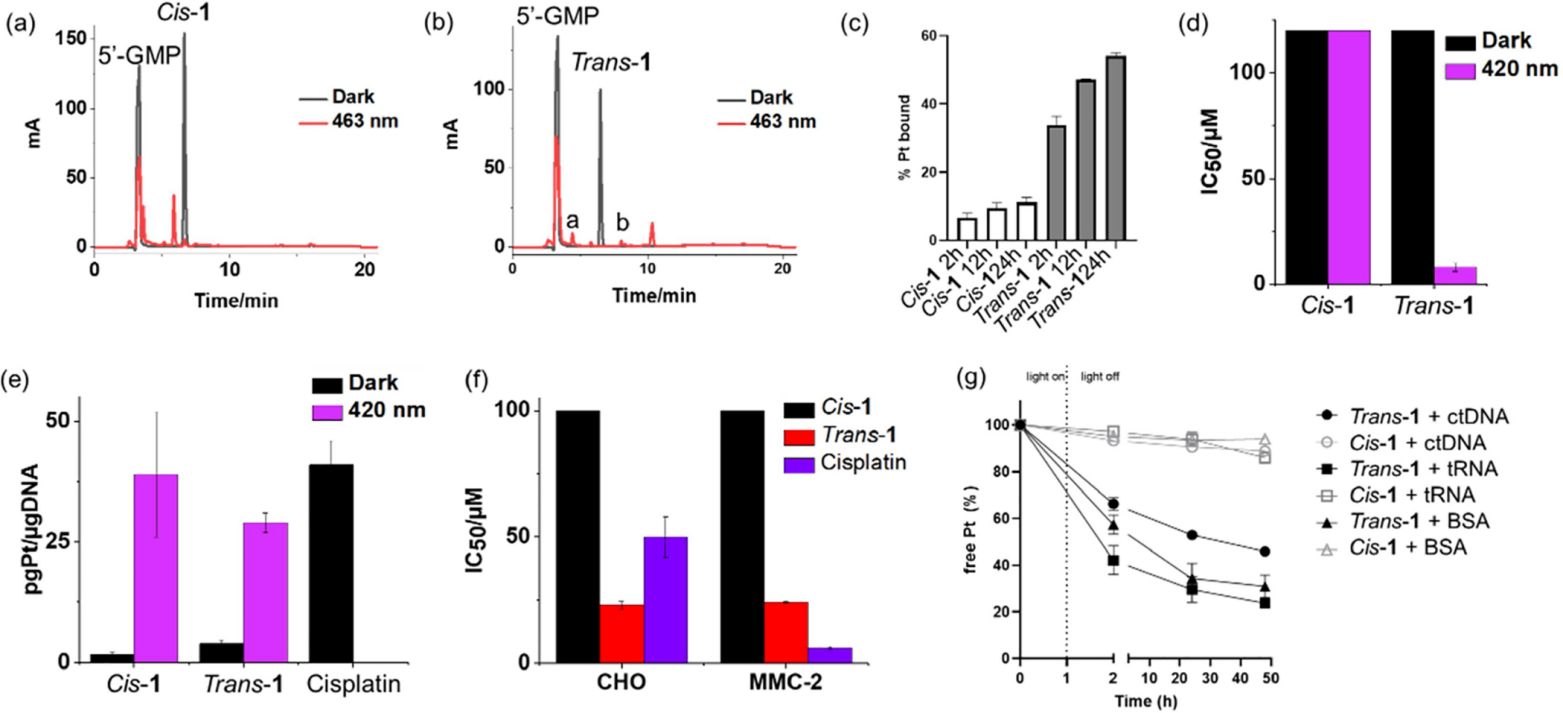
Photoreactions between complexes (a) *Cis*-**1** or (b) *Trans*-**1** and 2 mol. equiv of 5′-GMP after 1 h irradiation with blue light (463 nm), monitored by HPLC (at 254 nm), product a is [Pt­(MNZ)_2_(GMP)­(HCOOH)­(N_3_)]^+^ (*m*/*z* 972.05) and b is [Pt­(MNZ)_2_(CH_3_CN)­(OH)­(GMP-H)]^+^ (*m*/*z* 942.12); (c) binding of complexes to ctDNA after irradiation with blue light (465 nm); (d) IC_50_ values for complexes in SW780 bladder cancer cells after 1 h incubation, 1 h irradiation (indigo 420 nm) and 70 h recovery; (e) Pt content (in pg Pt/μg DNA) associated with DNA in SW780 bladder cancer cells; (f) IC_50_ values for complexes in wild-type CHO-K1 and mutant MMC-2 Chinese hamster ovarian cancer cells after 1 h incubation, 1 h irradiation (indigo 420 nm), and 70 h recovery; and (g) time-dependent Pt binding to ctDNA, tRNA, and BSA after incubation and irradiation with blue light (465 nm) for 1 h.

Both complexes showed a similar amount of Pt binding to nuclear DNA in SW780 bladder cancer cells upon irradiation (420 nm), despite their significantly different photocytotoxicity under the same conditions (Figure 5d,e). This suggested that either DNA is not the major target site, and/or the nature of the structural lesions is different. The pair of Chinese hamster ovary CHO-K1 cell lines, wild-type and its mutant line MMC-2 which is nucleotide excision repair (NER)-deficient carrying the ERCC3/XPB mutation, were used to distinguish whether NER-reparable DNA damage induced by these isomers is involved in the mechanism of action. MMC-2 cells are distinctly more sensitive to cisplatin, due to its mechanism involving nuclear DNA damage. In contrast, *Trans*-**1** showed no difference in sensitivity toward mutant and wild-type cell lines, suggesting that DNA damage plays a markedly less decisive role in the mechanism of its antiproliferative activity (Figure 5f).

Interstrand-cross-links (IECs) are known to be highly lethal and are not simply repaired by NER, but only 1.3 and 1% of IECs/Pt were detected for *Cis*-**1** and *Trans*-**1**, respectively (Figure S18). In comparison, cisplatin formed ca. 6% IECs per Pt adduct. In addition, both complexes induced only slight conformational distortions of DNA, based on the inhibition of EtBr intercalation into platinated DNA and the measurement of Tb^3+^ fluorescence, suggesting that DNA is not the main target for these isomers (Figure S19). Previously, adducts of photoactivated diazido Pt­(IV) complexes with biomolecules other than DNA have been identified.^48,49^ Thus, the time-dependent platination of ctDNA, tRNA and bovine serum albumin (BSA) after incubation and irradiation with 420 nm light for 1 h was studied for these isomers (Figure 5g). As seen in Figure 5g, very little Pt binding for *Cis*-**1** was observed even after 50 h. In contrast, there was significant binding of Pt from *Trans*-**1** to ctDNA (ca. 70%), greater than that to BSA (ca. 50%), but less than for tRNA (ca. 80%).

### ROS Generation and Lipid Peroxidation

Singlet oxygen generation was detectable for both isomers by trapping with TEMP to form TEMPO as a 1:1:1 EPR triplet (Figure S20). Cumulative ROS are regarded as key species in the mechanism by which photoactive diazido Pt­(IV) complexes, causing cell death. DCFH-DA is oxidized to DCF and emits green fluorescence in the presence of ROS. SW780 bladder cancer cells treated with 10, 20, and 50 μM *Cis*-**1** and *Trans*-**1** exhibited ca. 1.1–1.3× enhanced fluorescence (λ_ex_/λ_ex_ = 485/521–539 nm) upon irradiation with blue light (465 nm) compared those in the dark (Figure S21). The fluorescence intensity increased with concentration of the complexes. When cells were treated with irradiated complexes at 50 μM, the ROS levels reached those for cells treated with 5 μM H_2_O_2_. Notably, the antioxidant *N*-acetyl-l-cysteine can significantly scavenge ROS and therefore quench the fluorescence induced by the irradiated complexes. Lipid peroxidation is a hallmark of ferroptosis caused by accumulation of ROS.^50–52^ No apparent lipid peroxidation was observed for SW780 bladder cancer cells treated with 20 μM *Cis*-**1** or *Trans*-**1** upon irradiation (465 nm, Figure S22).

## Discussion

The differences between the chemical and biological activities of isomers of metal complexes have attracted much attention ever since the discovery that cisplatin is a highly active anticancer drug but transplatin is not.^1,2,13^ Understanding these differences can contribute to the design of novel metallodrugs, as well as elucidation of their mechanism of action.

In contrast to many square-planar cisplatin diamine analogues and their corresponding oxidized octahedral Pt­(IV) forms containing additional axial ligands, e.g. hydroxides, photoactive diazido Pt­(IV) prodrugs with all *trans*-configurations are more photocytotoxic than their *cis*-isomers, with red-shifted and more intense LMCT absorption bands.^31,32^ The reasons behind these differences are elucidated in the current study.

Octahedral photoactive *cis*- and *trans*-diazido dihydroxido Pt­(IV) isomers bearing metronidazole ligands were synthesized, characterized by X-ray crystallography and other methods, and the differences in photochemical and photobiological properties were determined experimentally and theoretically (Table 2). Metronidazole is a nitroimidazole and a drug widely used for treating anaerobic bacterial and antiprotozoal infections.^34,53^ Its activity relies on partial reduction and has little effect on either aerobic bacterial or human cells.^54^

**Table 2:** Comparisons between the Properties of *Cis*-**1** and *Trans*-**1** Isomers

property	*Cis*-1	*Trans*-1
Pt^IV^/ Pt^II^ reduction potential (V)	–1.27	–1.28
dark stability	high	high
λ_max_ (nm)	290	297
ε (M^–1^ cm^–1^)	21,389	31,818
triplet excited states	dissociative	dissociative
photoactivation rate	slower	quicker
azidyl radical formation	not detectable	yes
metronidazole ligand release	yes	no
dark cytotoxicity	low	low
photocytotoxicity	low	high
cellular accumulation	lower	higher
photoinduced apoptosis	no	yes
photoinduced mitochondrial membrane potential change	small	significant
photoinduced 5′-GMP binding	no	yes
photoinduced ctDNA, tRNA, BSA binding	no	yes
photoinduced cellular DNA binding	yes	yes
ability to circumvent NER	unknown	yes
photoinduced ROS generation	yes	yes
photoinduced lipid peroxidation	no	no

Both isomers exhibited negative potential electrochemical reduction waves, which correlates with their high dark stability. A red-shifted and ca. 50% more intense UV absorption band was observed for *Trans*-**1**, which was assigned to spin-allowed mixed LLCT, LMCT and LC excitations, suggesting more favorable photoreduction. In contrast, the absorption maximum of *Cis*-**1** is attributable to mixed LC and LLCT transitions, and absorption bands in the range from 255 to 456 nm involve LMCT transitions, which may make its changed spectra located in a blue-shifted wavelength range compared with those for *Trans*-**1** that was centered at the absorption maximum. Notably, metronidazole ligands play a more important role in the molecular orbitals of *Cis*-**1**, which might account for the photorelease of metronidazole from *Cis*-**1** only.

*Trans*-**1** was photocytotoxic toward SW780 bladder cancer cells with blue and green light irradiation under both normoxia and hypoxia, while *Cis*-**1** displayed no apparent photocytotoxicity under the same conditions. Generally, Pt­(II) photoproducts, azidyl radicals and ROS are thought to be the main cytotoxic photoproducts of diazido Pt­(IV) complexes.^27,28,40,48,55^

Pt­(II) species which bind to nucleotide bases in DNA and amino residues in proteins are reported as photoproducts of diazido Pt­(IV) anticancer complexes.^48,56,57^ The Pt­(II) species from *Trans*-**1** arise from photorelease of two azide ligands and contain the [Pt^II^(MNZ)_2_]^2+^ fragment, which has a high affinity for biomolecules, including 5′-GMP, ctDNA, tRNA and BSA in cell free media. In contrast, the Pt­(II) photoproducts from *Cis*-**1** involve photorelease of one azido and one metronidazole ligand from *trans* positions, and their binding abilities are much weaker compared with those from *Trans*-**1**. However, DNA isolated from cells treated with irradiated inactive *Cis*-**1** contains a similar amount of Pt as DNA from cells treated with active *Trans*-**1**, despite Pt cellular accumulation from irradiated *Trans*-**1** being >2× higher than that of *Cis*-**1**. This might be attributable to the binding of irradiated *Trans*-**1** to other biomolecules before it reaches nuclei. In addition, only ca. 1% formation of DNA interstrand cross-links and slight conformational distortion of DNA were induced by both isomers. This contrasts with the DNA interstand cross-links formed by photoactivated **FM190**, indicating the effect of the *N*-heterocyclic ligands on the DNA binding ability of photoactive Pt­(IV) complexes.^58^ Also, *Trans*-**1** showed no difference in potency toward the NER-deficient mutant and wild-type cell lines. These results indicated that damage to biological molecules other than DNA may also significantly contribute to the photoactivity of *Trans*-**1**. Although in a cell-free environment, *Trans*-**1** binds to DNA significantly more than *Cis*-**1**, *Trans*-**1** can already bind to potential targets in the cytoplasm with less of it reaching the nuclear DNA in cells.

Other than Pt­(II) species, azidyl radicals are important cytotoxic species released from diazido Pt­(IV) complexes upon irradiation.^40^ The formation of azidyl radicals was observed from *Trans*-**1** only after irradiation. TDDFT calculations suggest that two azidyl radicals are readily released from *Trans*-**1**, while only one azidyl radical is released from irradiated *Cis*-**1**, followed by the release of an MNZ ligand. The consequent reactions with Pt­(II) species in the triplet state might quench the azidyl radicals from *Cis*-**1** as indicated by DFT calculations. This will lead to a different sequence of redox (formation of Pt­(III) and Pt­(II) species and hydroxyl radical) and ligand substitutions that can contribute to the difference in photocytotoxicity between the two isomers. Photoinduced ^1^O_2_ generation in solution was observed by EPR for both isomers, which might result from H_2_O_2_ formed by the release of the two axial hydroxido ligands.^55^ Elevated ROS levels were observed in cells treated with either isomer after irradiation, but without apparent lipid peroxidation. ROS damage to intracellular targets such as proteins might be specific for the isomers and their photoproducts, which requires further investigation.

Significant mitochondrial membrane potential changes were observed when cells were treated with *Trans*-**1** and light, while *Cis*-**1** induced only mild changes, which is consistent with the difference in their photocytotoxicity. It seems likely that mitochondria are important targets for the cytotoxic species, Pt­(II) and azidyl radicals, from irradiated *Trans*-**1**, which leads to apoptotic cell death.

Detecting and identifying the photoproducts resulting from photoirradiation of these diazido-Pt­(IV) prodrugs is challenging on account of the multiple reaction pathways available, including photosubstition, photoreduction to Pt­(III) and Pt­(II), and radical formation. LC-MS studies can provide insight from reactions carried out at physiologically relevant micromolar Pt concentrations. However, there are clear limitations that must be borne in mind. First, HPLC reverse-phase compatibility and optimum separation of species require the use of less polar solvents together with an ion-pairing agent such as formate. Second, MS detects charged species only and those that enter the gas phase after passing through a capillary, high voltage an electric field. This often results in loss of ligands from metal complexes and sometimes redox reactions.

For *Cis*-**1** but not *Trans*-**1** on photoirradiation, loss on an amine ligand MNZ is observed for a photoreduced Pt­(II) product (Table S9). This amine-loss behavior is very unusual in comparison also with 3 related complexes we have studied previously (Am_1_/Am_2_ = pyridine/NH_3_ (**FM165**); Am_1_ = Am_2_ = pyridine (**FM190**) or 1-methyl-5-nitroimidazole (**HS28**)), but detectable for complexes with two *trans* 3-nitropyridine (MS data summarized in Table S22). The formation of 3 potentially reactive sites on Pt­(II) in comparison with only 2 for *Trans*-**1** and the other five complexes, is likely to allow the *Cis*-**1** photoproducts to be diverted from target sites which lead to cell death to other biomolecules.

Pt­(III) photoproducts were detected only for *Trans*-**1**, suggesting they arise from pathways not available to *Cis*-**1**, perhaps because of the facile loss of an MNZ ligand. Although Pt­(III) centers are well-known in ligand-bridged bi- and multinuclear complexes,^59,60^ they are usually highly unstable as monomeric complexes. Hence, although Pt­(III) photoproducts could result from photoinduced ligand-to-metal one-electron transfer to Pt­(IV), they would not be expected to survive in HPLC separations. Bednarski and co-workers observed Pt­(III) products by LC-MS after UVA (366 mm) irradiation of all-*trans*-[Pt­(N_3_)_2_(OH)_2_(py)­(NH_3_)] and discussed the possibility of these arising from redox reactions at the MS metal electrospray capillary electrode.^61^

Pt­(IV) substitution products were not separated for neither *Cis*-**1** nor *Trans*-**1**, although they are common for related diazido complexes (Tables S9 and 10).^62^ Polyhydroxido species in particular may be much more reactive than their parent dihydroxido complexes. Significant amounts of Pt­(IV) species are detectable by X-ray fluorescence mapping after visible light irradiation of, for example, **FM190** in PC3 prostate cancer cells, and might play a role in the mechanism of action.^63^

## Conclusions

Phototherapy provides precise tumor localization, low invasiveness and novel mechanisms of action to circumvent cross-resistance with current therapies for cancer treatment. With rich diversity in geometries, oxidation states, and coordination numbers, metallodrugs have attracted intense attention as potential photoactive drugs.^21,64–72^ We have synthesized and characterized two *cis*- and *trans*-photoactive diazido Pt­(IV) isomers bearing metronidazole ligands, and confirmed their configurations by single crystal X-ray crystallography.

Only *Trans*-**1**, and not its *cis* isomer, was active toward bladder cancer cells, including under hypoxia. A red-shifted and 50% more intense LMCT band was observed for *Trans*-**1**, in which azido ligands play a more important role in the molecular orbitals as indicated by computational analysis. Both isomers display a dissociative triplet state, which promotes reduction upon irradiation. Photoreleased azidyl radicals were detected for *Trans*-**1**, while metronidazole was readily released only from *Cis*-**1**. Such findings are in very good agreement with the outcomes of DFT calculations. Photoreduced Pt­(II) species from *Trans*-**1** react with biomolecules more strongly than those from its *cis* isomer. RNA and proteins in the cytoplasm, rather than nuclear DNA, may be the main targets of these species in cells. Azidyl radicals and Pt­(II) species were assigned as the main cytotoxic species released from these isomers upon irradiation, which result in differences in their photocytotoxicity. While ROS generation may not behave as the main factor which contributes to the mechanism of these isomers, both isomers generate similar levels of photoinduced ROS, also both isomers induce low levels of lipid peroxidation upon irradiation. Mitochondrial damage and apoptosis were observed only for cells treated with *Trans*-**1** and light.

This study has explored the excited state metallomics of *cis* and *trans* diamine diazido Pt­(IV) anticancer isomers, identifying reactive species formed during photoactivation pathways that could be involved in their mechanism of action, and explaining their contrasting anticancer cytotoxicity, opposite to traditional structure–activity relationships for the early ground-state platinum anticancer drugs. The new mechanisms of action proposed here can be incorporated into future designs of photoactive targeted Pt­(IV) anticancer drugs with improved efficacy and reduced side-effects.

## Experimental Section

### Synthesis and Characterization

*Caution!* We encountered no problem during this work, but due care and attention with appropriate precautions should be taken in the synthesis and handling of shock-sensitive heavy metal azides in the dark.

### *Cis*, *cis*, *trans*-[Pt­(N_3_)_2_(metronidazole)_2_(OH)_2_] (*Cis*-**1**)

A solution solution of K_2_PtCl_4_ (200 mg, 482 μmol) with 2 mol. equiv of metronidazole (165 mg, 964 μmol) in H_2_O (2 mL) was stirred at 323 K for 12 h, followed by 363 K for 12 h. Then 20 mol. equiv of NaN_3_ (628 mg, 9662 μmol) in 4 mL H_2_O was added dropwise to the resulted mixture, and stirred for 24 h at 363 K. The yellow precipitate (174 mg) was filtered off, washed with H_2_O and EtOH, then suspended in 15 mL H_2_O_2_, then stirred at 318 K for 12 h giving a yellow solution. The solution was filtered and lyophilized by freeze-drying, and purified on a Biotage Sfär C18 D column. Yield: 42 mg, 13%. HPLC purity: 99.7%. ^1^H NMR (DMSO-*d*_6_, 500 MHz): 8.16 (s with Pt satellites, *J* ^195^Pt–^1^H = 15.26, 2H, *H*_a_), 5.09 (t, *J* = 5.30, 2H, *OH*), 4.46 (t, *J* = 5.00, 4H, *CH*_*2*_), 3.72 (q, *J* = 5.16, 4H, *CH*_*2*_), 2.58 (s, 6H, *CH*_*3*_), 1.38 (s, 2H, *OH*). ^13^C NMR (DMSO-*d*_6_, 125 MHz): 153.40, 138.19, 130.07, 59.64, 49.72, 12.07. ESI-HR-MS: [M + Na]^+^ (*m*/*z*) Calc., 678.1067; Found, 678.1066.

### *Trans*, *trans*, *trans*-[Pt­(N_3_)_2_(metronidazole)_2_(OH)_2_] (*Trans*-**1**)

An H_2_O solution (2 mL) of K_2_PtCl_4_ (200 mg, 482 μmol) with 8 mol. equiv of metronidazole (660 mg, 3856 μmol) was stirred at 363 K for 24 h. Them the solvent was removed and the residue washed with ether. Six mL of 2 M HCl was added to the residue and stirred for another 24 h. After filtration, a light yellow solid (262 mg) was obtained which was washed with H_2_O and EtOH. The solid was mixed with 20 mol. equiv of NaN_3_ (552 mg, 8.5 mmol) in 10 mL DMF, and the mixture was stirred for 24 h at 298 K. The brown precipitate (89 mg) was filtered off, washed with H_2_O and EtOH, suspended in 15 mL H_2_O_2_, and stirred at 318 K for 24 h to give a yellow solution. The solution was filtered and lyophilized by freeze-drying, then purified on a Biotage Sfär C18 D column. Yield: 16 mg, 5%. HPLC purity: 100%. ^1^H NMR (DMSO-*d*_6_, 500 MHz): 8.31 (s with Pt satellites, *J* ^195^Pt–^1^H = 17.78, 2H, *H*_a_), 5.19 (t, *J* = 5.38, 2H, *OH*), 4.58 (t, *J* = 4.91, 4H, *CH*_*2*_), 3.79 (q, *J* = 5.16, 4H, *CH*_*2*_), 3.04 (s, 6H, *CH*_*3*_), 1.60 (s, 2H, *OH*). ^13^C NMR (DMSO-*d*_6_, 125 MHz): 153.34, 137.58, 129.95, 59.74, 49.82, 12.83. ESI-HR-MS: [M + Na]^+^ (*m*/*z*) Calc., 678.1067; Found, 678.1067.

## Supplementary Material



## Notes

Additional experimental information, materials, instruments; relevant tables, scheme, and figures; cell culture, cytotoxicity, cell Pt accumulation, and distribution; ROS assays, DNA and protein binding, DNA repair, DFT calculations, TTDFT data, natural transition orbital crystallographic data, CV data, LC-MS data, IC_50_ values and photocytotoxicity indices, HPLC chromatograms, photoproducts, HR-ESI-MS, and NMR; EPR and UV–vis spectra, excited state reaction mechanisms, membrane potentials, apoptosis, and lipid peroxidation (PDF)
