# Supplementary material for: Excited-State Cis and Trans Pt(IV) Diamine Anticancer Complexes
Source: Inorg Chem. 2025 May 28;64(22):11301–11. doi: 10.1021/acs.inorgchem.5c01882 (PMC12152928; doi:10.1021/acs.inorgchem.5c01882)
Supplement: Supplementary file 1 [file ic5c01882_si_001.pdf]

Supporting Information

Excited-state *cis* and *trans* Pt(IV) diamine anticancer complexes

Huayun Shi,[a,b*] Jana Kasparkova,[c,d] Fortuna Ponte,[e] Hana Kostrhunova,[d] Guy J. Clarkson,[a] Emilia Sicilia,[e*] Viktor Brabec [c,d*] and Peter J. Sadler[a*]

[a] Department of Chemistry, University of Warwick, Coventry CV4 7AL, United Kingdom

[b] State Key Laboratory of Vaccines for Infectious Diseases, Xiang An Biomedicine Laboratory, National Innovation Platform for Industry-Education Integration in Vaccine Research, School of Public Health, Xiamen University, Xiamen 361102, China

[c] Department of Biophysics, Faculty of Science, Palacky University, CZ-77900 Olomouc, Czech Republic

[d] Czech Academy of Sciences, Institute of Biophysics, Czech Academy of Sciences, CZ-61200 Brno, Czech Republic

[e] Department of Chemistry and Chemical Technologies, University of Calabria, via Pietro Bucci, 87036 Arcavacata di Rende, Cs, Italy

Email: huayun.shi@xmu.edu.cn; emilia.sicilia@unical.it; vbrabec44@gmail.com; p.j.sadler@warwick.ac.uk

Contents

Experimental section. 5

Table S1. Crystal data and structure refinement for *Cis*-**1** and *Trans*-**1**·2MeOH. 14

Table S2. Selected bond lengths (Å) and bond angles (˚) for *Cis*-**1** and *Trans*-**1**·2MeOH..... 15

Table S3. Hydrogen bonds for *Cis*-**1** and *Trans*-**1**·2MeOH. 15

Table S4. TDDFT benchmark for the reproduction of the experimental spectrum of *Cis*-**1** on the structure optimized at B3LYP-D3/6-31G* level in H_2O implicit solvent, λ_{exp} = 290 nm. 16

Table S5: B3LYP-D3 excitation energies (ΔE, eV), absorption wavelength (λ, nm), oscillator strength (*f*), MO contribution (%) for selected transitions Tr for *Cis*-**1** and *Trans*-**1**. 17

Table S6. Natural Transition Orbitals (NTOs) for the most important transitions of *Cis*-**1**. . 19

Table S7. NTOs for the most important transitions of *Trans*-**1**. 20

Table S8. Cyclic voltammogram data for complexes and corresponding ligands. 20

Table S9. Observed and calculated masses of photoproducts of *Cis*-**1** (50 µM) after 1 h irradiation (463 nm) determined by LC-MS (mobile phase: water and acetonitrile). 21

Table S10. Observed and calculated masses of photoproducts of *Tras*-**1** (50 µM) after 1 h irradiation (463 nm) determined by LC-MS (mobile phase: water and acetonitrile). 21

Table S11. B3LYP-D3 excitation energies (ΔE, eV), MO contribution (%) and theoretical assignment for the singlet states of *Cis*-**1** and *Trans*-**1** involved in the ISC process, calculated by B3LYP functional. 21

Table S12. B3LYP-D3 excitation energies (ΔE, eV), MO contribution (%) and theoretical assignment for the triplet states of *Cis*-**1** and *Trans*-**1** located below the bright state calculated by B3LYP functional. 22

Table S13. NTOs for the singlet state (bright state) of *Cis*-**1**. 23

Table S14. NTOs for the singlet states below the bright state of *Trans*-**1**. 23

Table S15. NTOs for the triplet states of *Cis*-**1**. 23

Table S16. NTOs for the triplet states of *Trans*-**1**. 24

Table S17. Spin orbit coupling constants (SOC, cm^{-1}) between the bright state and triplet states lying below, together with their adiabatic energy difference (ΔE, eV) in parentheses, calculated for *Cis*-**1** and *Trans*-**1**. 24

Table S18. Most relevant bond lengths (Å) in *Cis*-T_a, *Cis*-T_b and *Trans*-T_a triplet structures together with spin density values (SD in a.u.) on the platinum metal center and the most important atoms of the coordinated ligands. 25

Table S19. IC_{50} values and photocytotoxic indices (PI) for complexes in A2780 and cisplatin-resistant A2780cis ovarian cancer cells after 1 h incubation, 1 h irradiation (blue 465nm, green 520 nm) and 72 h further incubation under normoxia (21% O_2). 25

Table S20. Cellular Pt accumulation from complexes (10 µM) in SW780 bladder cancer cells after 1 h incubation, 1 h irradiation (blue 465nm) under normoxia (21% O_2) and hypoxia (1% O_2). 26

Table S21. Cellular Pt distribution after incubation of SW780 bladder cancer cells with complexes (10 μM) for 2 h in the dark under normoxia (21% O_2). 26

Table S22. Comparison of photoproducts of diazido Pt(IV) complexes detected by LC-MS.27

Scheme S1. The synthetic routes for Pt(IV) complexes *Cis*-**1** (A) and *Trans*-**1** (B).............. 29

Figure S1. HPLC chromatograms for complexes *Cis*-**1** and *Trans*-**1**. 29

Figure S2. HR-ESI-MS of complexes (a) *Cis*-**1**and (b) *Trans*-**1** in positive mode................ 30

Figure S3. 500 Hz ^{1}H NMR spectrum of complex *Cis*-**1** in DMSO-d_6 at 298 K. 30

Figure S4. 125 Hz ^{13}C-{^{1}H} APT NMR spectrum of complex *Cis*-**1** in DMSO-d_6 at 298 K. 31

Figure S5. 500 Hz ^{1}H NMR spectrum of complex *Trans*-**1** in DMSO-d_6 at 298 K. 31

Figure S6. 125 Hz ^{13}C-{^{1}H} APT NMR spectrum of complex *Trans*-**1** in DMSO-d_6 at 298 K. 32

Figure S7. B3LYP-D3 optimized structure of *Cis*-**1** and *Trans*-**1** isomers along with the most relevant geometrical parameters. Bond lengths are in Å. 32

Figure S8. Cyclic voltammograms of complexes (a) *Cis*-**1**, (b) *Trans*-**1**, and (c) and metronidazole, (1 mM in 0.1 M NBu_4PF_6-DMF, under N_2)... 33

Figure S9. UV-vis spectra showing the dark stability of complexes *Cis*-**1** and *Trans*-**1** in air (a for *Cis*-**1**; b for *Trans*-**1**) and N_2-saturated (c for *Cis*-**1**; d for *Trans*-**1**) MillQ H_2O at 310 K for 2 h. 33

Figure S10. HPLC of (a) *Cis*-**1** and (d) *Trans*-**1**, 50 μM) in aqueous solution after incubation in the dark at 310 K for 0 and 24 h, detection at 254 nm. 33

Figure S11. UV-vis spectra showing the dark stability of complexes *Cis*-**1** and *Trans*-**1** (50 μM) in the presence of 2 mM GSH in MillQ H_2O at 298 K for 2 h. 34

Figure S12. Time dependent UV-vis spectral changes for complexes *Cis*-**1** and *Trans*-**1** in N_2-saturated MillQ H_2O exposed to blue (463 nm, (a) *Cis*-**1**; (b) *Trans*-**1**) or green (517 nm, (d) *Cis*-**1**; (e) *Trans*-**1**) light at 298 K; (c) and (f), plots of time-dependent absorbance changes for complexes at the absorption maximum (290 nm for *Cis*-**1**; 297 nm for *Trans*-**1**) upon irradiation with light of different wavelengths ((c) 463 nm; (f) 520 nm). 34

Figure S13. HPLC for 50 μM complex in water, (a) *Cis*-**1** and (b) *Trans*-**1** in the dark (black traces) and after 1 h irradiation with blue light (463 nm). Mass spectra of photoproducts (c) c1–c2 and (d) t1–t5 are presented and the possible assignments are listed in Tables **S9** and **S10**.35

Figure S14. Proposed excited triplet state reaction mechanisms for the formation of (a) the photoproducts *cis*-[$Pt^{II}(MNZ)(OH)(H_2O)_2]^+$ and (b) *trans*-[$Pt^{II}(MNZ)_2(H_2O)(N_3)]^+$. 36

Figure S15. Mitochondrial membrane potentials analysed by flow cytometry for SW780 cells under normoxia treated with *Cis*-**1** and *Trans*-**1** (a for 10 μM and b for 20 μM) in the dark (2 h) or 1 h incubation and 1 h irradiation (465 nm), and 72 h further incubation, then stained by TMRE ($\lambda_{ex}/\lambda_{em}$ = 561/570–600 nm). 37

Figure S16. Mitochondrial membrane potential analysed by flow cytometry for SW780 cells under hypoxia treated with (a for 10 μM and b for 20 μM) *Cis*-**1** and *Trans*-**1** in the dark (2 h) or 1 h incubation and 1 h irradiation (465 nm), and 72 h further incubation, then stained by TMRE ($\lambda_{ex}/\lambda_{em}$ = 561/570–600 nm). 37

Figure S17. Cell apoptosis assays for SW780 cells under normoxia double-stained by Annexin V-FITC/PI ($\lambda_{ex}/\lambda_{em}$ = 488/500–560 nm for Annexin V-FITC, $\lambda_{ex}/\lambda_{em}$ = 488/645–735 nm for PI) and analyzed by flow cytometry. a) untreated SW780 cells in the dark and d) irradiated with blue light (465 nm); b) SW780 cells treated with *Cis*-**1** (20 µM) in the dark and e) irradiated with blue light; c) SW780 cells treated with *Trans*-**1** (20 µM) in the dark and f) irradiated with blue light. 38

Figure S18. Autoradiograms of denaturing 1% agarose gel of linearized pDNA; the interstrand cross-linked DNA appears as the top bands (IECs) migrating on the gel more slowly than the single-stranded (ss) DNA (contained in the bottom bands). Plasmid linearized by EcoRI was incubated for 24 h with cisplatin, *Cis*-**1**, and *Trans*-**1** at r_b values of 0 (control, lanes 1 and 8), 0.0005 (lanes 2, 4, 6) or 0.001 (lanes 3,5,7) (r_b= number of Pt bound per nucleotide residue). 38

Figure S19. (a) Plots of EtBr fluorescence versus r_b for DNA modified by Pt-complexes for 24h (1 h irrad 465 nm + 23 h in the dark for *Cis*-**1** and *Trans*-**1**, samples with cisplatin were incubated in the dark for 24 h). (b) Change in the relative fluorescence of Tb^{3+} ions bound to double-helical *ct*DNA modified by platinum complexes. The fluorescence of the untreated DNA-EtBr was arbitrarily set at 1. 39

Figure S20. EPR spectra for complexes (2.5 mM) (a) *Cis*-**1** and (b) *Trans*-**1** in the presence of TEMP (20 mM) in acetonitrile (containing 5% DMSO) to trap singlet oxygen; Dark (—); blue light (—, 463 nm, 20 min). The experimental trace is for accumulation of 5 scans (conversion time 10.24 ms, time constant 10.24 ms, and sweep time 20.97 s for each scan)........ 39

Figure S21. Relative fluorescence intensity of SW780 cells treated with *Cis*-**1** or *Trans*-**1** (2 h or 1 h in the dark and 1 h irradiation, 465 nm) in the (a) absence and (b) presence of 10 mM antioxidant *N*-acetyl-L-cysteine (NAC), then probed with DCFH-DA (20 µM, λ_{ex} = 485 nm, λ_{em} = 521–539 nm). 40

Figure S22. Lipid peroxidation assay for SW780 cells stained by BODIPY™ 581/591 C11 ($\lambda_{ex}/\lambda_{em}$ = 488/500–560 nm) and analyzed by flow cytometry. 40

Experimental section.

Materials and Instruments. Metronidazole (MNZ) was purchased from Thermo Scientific Chemicals, K_2PtCl_4, NaN_3, H_2O_2 (30%) and other chemicals were from Sigma Aldrich and used without further purification. Calf thymus DNA (ctDNA) and yeast transfer RNA (tRNA) were from Merck and Invitrogen, respectively. Plasmid pUC19 (2686 base pairs), fatty acid-free bovine serum albumin (BSA), DNA kinase, and restriction endonuclease EcoRI were purchased from New England Biolabs (Beverly, MA). Agarose was from FMC BioProducts (Rockland, ME, USA). [gamma-^{32}P]ATP was from Hartmann Analytic GmbH, Braunschweig, Germany.

NMR spectra were recorded on a Bruker Avance III HD 500 MHz spectrometer using residual signal of the solvent DMSO-d_5 as a reference in ^{1}H NMR and that of DMSO-d_6 in ^{13}C NMR. ESI-MS spectra were recorded on an Agilent 6130B single quadrupole detector instrument at 298 K with a scan range of m/z 50-2000 for positive ions and HR-MS data were collected on a Bruker microTOF instrument at 298 K.

Electronic absorption spectra were recorded on a Varian Cary 300 UV-vis spectrophotometer using a quartz cuvette and solvent as reference. The spectral width was 200–600 nm and the bandwidth 1.0 nm, scan rate 600 nm/min.

LC-MS was carried out using a Bruker Amazon X mass spectrometer connected online to an Agilent 1260 HPLC with an Agilent ZORBAX Eclipse XDB-C18 column (250×4.6 mm, 5 μm, flow rate: 1 mL/min), using linear gradients of 0.1% FA in H_2O (solvent A) and 0.1% FA in CH_3CN.

The light sources used for photoactivation were LED light sources (BASETech model no. SP-GU10 230 V~50 Hz 1.3-2.1 W) with λ_{max} = 463 or 517 nm. A 96-array of LEDs with λ_{max} = 465 (4.8 mW cm^{-2} per LED) or 520 (11.7 mW cm^{-2} per LED) nm was used for *in vitro* growth inhibition. Whitley miniMACS Anaerobic Workstation equipped with a BOC gas cylinder (1% O_2/5% CO_2/94% N_2, 200 bar) was used to provide the hypoxia environment for *in vitro* growth inhibition.

Platinum contents were analyzed on an ICP-MS 7900 (Agilent), ICP-OES 5300DV (Perkin Elmer) or ICP-OES 5800 (Agilent). The emission wavelength detected for Pt in ICP-OES was 265.945 nm.

X-Ray crystallography. Light yellow single crystals of *Cis*-**1** and *Trans*-**1**·2MeOH were grown by slow evaporation of methanol solutions. Suitable crystals were selected and mounted on a glass fiber with Fomblin oil and placed on a Rigaku Oxford Diffraction SuperNova diffractometer with a dual source (Cu at zero) equipped with an AtlasS2 CCD area detector.

The crystal was kept at 150(2) K during data collection. Using Olex2,[1] the structures were solved with the ShelXT[2] structure solution program using Intrinsic Phasing and refined with the ShelXL[3] refinement package using Least Squares minimization.

HPLC method. The injection volume was 50 µL of solution for each HPLC system. The gradient elution is shown below.

Time/min	Mobile Phase B/%
0	10
10	30
15	30
16	10
21	10

Electrochemistry. All cyclic voltammogram (CV) experiments were carried out using a CH Instrument model 600D Electrochemical Analyzer/Workstation (Austin, TX). The complex (1 mM) solution was prepared in DMF containing tetrabutylammonium hexafluorophosphate (0.1 M) as supporting electrolyte and degassed under nitrogen. A typical three-electrode system was used to scan the cyclic voltammograms: a glassy carbon electrode as the working electrode, Ag/$AgNO_3$ (10 mM in DMF) as the reference electrode, and platinum wire as the counter electrode. The scan rate was set to be 100 mV/s.

Dark stability and photoactivation in aqueous solution. The dark stability at 310 K and photoactivation at 298 K of complexes (50 µM) in air or N_2 saturated MilliQ water were monitored by UV-vis. The dark stability in the presence of 2 mM GSH in MilliQ water was monitored by UV-vis. The dark stability at 310 K and photoactivation at different time intervals upon irradiation with blue light (463 nm) at 298 K in aqueous solution were also determined by LC-MS.

Electron paramagnetic resonance (EPR) spectroscopy. The EPR spectra were recorded on a Bruker EMX (X-band) spectrometer at 298 K. Samples (*ca.* 100 µL) in aqueous solution were prepared and transferred using a plastic syringe with metal needle to a standard quality quartz tube with inner diameter of 1.0 mm and outer diameter of 2.0 mm (Wilmad LabGlass) and sealed with parafilm. Using the y-incremental sweep mode of 100 with an accumulation of 5 scans in the x dimension. Typical key EPR spectrometer settings were modulation amplitude 1.0 G, microwave power 6.31 mW, receiver gain 1.0×10^5, conversion time 10.24 ms, time constant 10.24 ms, sweep width 200 G. The blue LED (463 nm) fitted into a GU-10 device was

mounted within the EPR magnet, supported by a foam sponge, to maintain its position throughout the EPR measurements. The distance from the tip of the irradiation light bulb to the EPR cavity was *ca*. 5 cm. Data were processed by Matlab R2021b with easyspin 5.2.35.

Cell culture. Human cell lines, bladder cancer SW-780 cells, ovarian carcinoma A2780 and cisplatin-resistant A2780cis cells, were obtained from the European Collection of Animal Cell Culture (ECACC), Salisbury, UK. Chinese hamster ovary CHO-K1 cell line (wild type) and its derivative MMC-2 carrying the ERCC3/XPB mutation (NER-deficient) cell line were kindly supplied by Dr. M. Pirsel, Cancer Research Institute, Slovak Academy of Sciences, Bratislava (Slovakia). A2780 and A2780cis cell lines were grown in Roswell Park Memorial Institute media (RPMI-1640, Sigma Aldrich) supplemented with 10% v/v of heat-inactivated fetal bovine serum (Sigma Aldrich) and 1% v/v penicillin/streptomycin. While SW-780, CHO-K1 and MMC-2 cell lines were grown in DMEM medium (high glucose, 4.5 gL^{-1}, PAA) supplemented with 10% v/v heat-inactivated fetal bovine serum and 1% v/v penicillin/streptomycin. The adherent monolayers of cells were grown at 310 K in a humidified atmosphere containing 5% CO_2 and passaged regularly at *ca*. 80% confluence.

Photo-dark cytotoxicity under normoxia and hypoxia. Approximately 1×10^4 cells were seeded per well in 96-well plates. Independent duplicate plates were used, one for dark while the other for irradiation experiment. The cells were pre-incubated in drug-free medium with phenol red at 310 K for 24 h. For normoxia experiments, drugs were added after pre-incubation, while for hypoxia experiments, plates were transferred to hypoxia chamber and allowed another 24 h incubation under the hypoxia environment; all the other experiments were carried out under hypoxia. Drug treatments under these two conditions are exactly same, except for the oxygen concentration. Complexes were dissolved first in DMSO (complexes show high dark stability in DMSO) and then diluted in phenol red-free medium to make the stock solution of the drug. These stock solutions were further diluted using phenol-red free cell culture medium until working concentrations were achieved, with the DMSO concentration adjusted to be 0.05% v/v in these solutions. Cells were exposed to drugs with different concentrations for 1 h. Then one plate was irradiated for 1 h using blue light (4.8 mW cm^{-2} per LED at 465 nm) or green light (11.7 mW cm^{-2} per LED at 520 nm), while the dark plate was kept in the incubator. After irradiation, cells were incubated for another 72 h at 310 K under corresponding oxygen concentration without drug removal. Untreated controls that were only exposed to vehicle were also compared between the irradiated and the non-irradiated plates to ensure that the differences in cell survival were not statistically relevant, hence guaranteeing that the differences in cell viability observed were not due to the light source. The SRB assay was used to determine cell

viability.[4] Absorbance measurements of the solubilized dye (on a Tecan microplate reader) allowed the determination of viable treated cells compared to untreated controls. IC_{50} values (concentrations which caused 50% of cell death) were determined as the average of triplicates and their standard deviations were calculated. Stock concentrations for all metal complexes used in these biological assays were adjusted/verified after ICP-OES metal quantification.

Platinum accumulation in cancer cells. For Pt cellular accumulation studies, *ca*. 5×10^6 SW780 cells were plated in 100 mm Petri dishes and allowed to attach for 24 h. For normoxia experiments, drugs were added after pre-incubation, while for hypoxia experiments, plates were transferred to hypoxia chamber and allowed another 24 h incubation under hypoxia environment, and all the rest experiments were carried out under hypoxia. Drug treatments under these two conditions are exactly same except for the oxygen concentration. The plates were exposed to complexes at 10 μM in phenol red-free medium with 0.05% v/v DMSO. Additional plates were incubated with vehicle alone as a negative control. After 1 h of incubation in the dark at 310 K, one plate was left in the dark for another 1 h in the incubator, while the other one was irradiated with blue light (4.8 mW cm^{-2} per LED at 465 nm) or green light (11.7 mW cm^{-2} per LED at 520 nm). The cells were rinsed three times with cold PBS and harvested by trypsinization. The number of cells in each sample was counted manually using a hemocytometer. Then the cells were centrifuged to obtain the whole cell pellet for ICP-MS analysis. All experiments were conducted in triplicate.

Intracellular distribution. *ca*. 1×10^7 SW780 cells were plated in Petri dishes and allowed to attach for 24 h. Then the plates were exposed to complexes at 10 µM in the dark for 2 h at 310 K. Cells were rinsed three times with cold PBS and harvested by trypsinization. The number of cells in each sample was counted manually using a hemocytometer. Mitochondria/Cytosol Fractionation Kit were used to extract nuclei, mitochondria and cytoplasm. All experiments were conducted in triplicate.

ICP-MS sample preparation. Whole cell pellets in Eppendorf tubes were dissolved in concentrated 72% v/v nitric acid (200 μL), and heated in an oven at 343 K overnight. The samples were then allowed to cool, and each cellular sample solution was transferred into a Falcon tube and diluted with Milli-Q water (3.8 mL), to give a final HNO_3 concentration of *ca*. 3.6% v/v.

Flow Cytometry. All flow cytometry experiments were carried out using a Becton Dickinson FACScan flow cytometer in the School of Life Sciences at the University of Warwick. SW780

cells (*ca*. 5×10^5) were seeded in 6-well plates and cultured 24 h for attachment. Half of plates were moved to hypoxia chambers and left for 24 h and all the rest experiments were carried out under hypoxia. Drug treatments under these two conditions were exactly same except for the oxygen concentration. **Apoptosis assay.** Cells were exposed to complex for 1 h in dark then 1 h irradiation with blue light (465 nm), and further incubated with drugs for 72 h after irradiation, then washed with PBS and treated with Annexin V-FITC/PI kit (Abcam). **Lipid peroxidation assay.** Cells were exposed to complex (20 μM) for 1 h in the dark then 1 h irradiation with blue light (465 nm). Drugs were removed and cells were incubated with BODIPY™ 581/591 C11 (5 μM) for 30 min. Cells were washed by HBSS before measurement. **Mitochondrial membrane potential.** Cells were exposed to complex for 1 h in dark then 1 h irradiation with blue light (465 nm), and further incubated with drugs for 72 h after irradiation. Drugs were removed and cells were incubated with TMRE (200 nM) for 30 min. Cells were washed by PBS before measurement.

Intracellular ROS measurement. 2',7'-Dichlorofluorescin diacetate (DCFH-DA, Sigma-Aldrich) was used to determine the intracellular ROS production in the dark and after irradiation when SW780 cells were exposed to Pt(IV) complexes. SW780 cells were seeded in 96-well plates (*ca*. 10^4 cells per well, 24 h for attachment) then exposed to complexes at various concentration for 1 h. One plate was irradiated for 1 h with blue light (465 nm), while the other one was kept in the dark. Drugs were removed and DCFH-DA (20 μM) was added to cells and incubated for 30 min. Cells were washed by Hank's Balanced Salt Solution (HBSS). The fluorescence intensity was measured on a Tecan microplate reader with comparison to negative control.

Photoreactions with 5'-GMP. 2 mol. equiv. of guanosine 5′-monophosphate disodium salt hydrate (5′-GMP-Na_2) were mixed with 50 μM complex in aqueous solution. The solution was irradiated for 1 h (463 nm) and analyzed immediately by LC-MS.

Binding to biomacromolecules in cell-free media. The binding of complexes to ctDNA, tRNA, or BSA was assessed using UV-vis absorption spectroscopy. Pt complexes were mixed with ctDNA or tRNA in 0.01 M $NaClO_4$ ([Pt]/[nucleotide] = 0.1) or with BSA ([Pt]/[BSA] = 1). The per nucleotide concentration of both ctDNA and tRNA in the reaction mixture was 5.6 $\times$ 10^{-4} M, the concentration of BSA was 5.6 x 10^{-5} M. Samples were irradiated with LED centered at 465 nm (LED465E, Thorlabs) for 1 h and subsequently incubated in the dark at 310 K. After certain time intervals, aliquots were withdrawn, and biopolymers were removed by centrifugation through Nanosep® centrifugal devices (Nanosep 30 K for ctDNA and BSA,

Nanosep 10 K for tRNA). The free, unbound Pt-complex concentration in the filtrate was determined by UV-vis spectroscopy (Beckmann DU-7400 spectrophotometer). The absorbance of the sample, prepared and handled (including irradiation) in the same way but in the absence of DNA, RNA, and BSA, was taken as 100%.

Interstrand cross-link assay. Plasmid pUC19 DNA (0.5 µg) linearized by EcoRI and 5´-end-labeled by DNA kinase and [γ-^{32}P]ATP (exchange reaction) was incubated with Pt complexes at different concentrations; samples were irradiated for 1 h (465 nm) and further incubated at dark for 23 h. The concentrations of Pt complexes for incubation with DNA were chosen with regard to the results of the experiment quantifying the amount of Pt bound to DNA after 24 h of incubation so that the resulting amount of Pt bound to DNA was equivalent for all three tested complexes.

The number of interstrand cross-links (IECs) was analyzed by electrophoresis under denaturing conditions on alkaline agarose gel (1%). After the electrophoresis was completed, the gel was dried, exposed to a phosphor imaging plate, and scanned using a GE Healthcare FLA 7000 laser scanner. The intensities of the bands corresponding to single strands of DNA and IEC duplexes were quantified by Aida Image analysis software (Raytest, Germany). The frequency of IECs was calculated as %IEC/Pt = XL/5,372·r_b, where %IEC/Pt is the number of IECs per adduct, 5,372 is the number of nucleotide residues in pUC19 plasmid and r_b is defined as the number of molecules of the platinum complex bound per one nucleotide residue. XL is the number of IECs per molecule of the linearized DNA duplex and was calculated assuming a Poisson distribution of the IECs as XL = -ln A, where A is the fraction of molecules running as a band corresponding to the non-cross-linked DNA.

Characterization of DNA Adducts by EtBr Fluorescence. ctDNA was incubated for 24 h (1 h irradiation, 23 h dark) with various concentrations of the studied complexes. The concentrations were chosen considering the different binding capacities of the tested complexes so that it was possible to prepare samples with a preselected amount of Pt bound to DNA. Thus, the samples were comparable in terms of the amount of bound Pt. After incubation, the unbound molecules of Pt complexes were separated via centrifugation through Sephadex G25 columns, and DNA (10 ng/mL) was incubated with EtBr (40 ngmL^{-1}) in 0.4 M NaCl at 25 °C for 30 min. Fluorescence measurements were performed using a SPARK TECAN reader (SCHOELLER) in 96-well black plastic plates. An excitation wavelength was 546 nm, and the emitted fluorescence was analyzed at 590 nm. The high concentration of NaCl is used to avoid secondary binding of EtBr to DNA.[5]

Characterization of DNA distortion by terbium fluorescence. The DNA samples were prepared as described above (EtBr fluorescence measurement). Samples of ctDNA (8 μgmL^{-1}, 2.5 × 10^{-5} M) modified with Pt complexes to the preselected r_b were incubated with $TbCl_3$ (5 × 10^{-5} M) for 60 min at 25 °C in the dark. The fluorescence intensity was then measured on a Varian Cary Eclipse spectrofluorometer using a 1 cm quartz cell with λ_{ex} =290 nm and λ_{em} = 546 nm. More detailed information can be found in ref. 6.

DNA platination in cells. Pt content associated with DNA isolated from SW780 cells treated with Pt complexes was determined by ICP-MS. Cells were treated with 20 µM Pt complex in EBSS for 1 h in the dark. Then, some Petri dishes with cells were irradiated for 1 h using an LZC-4 photoreactor (Luzchem Research, Gloucester, Canada) equipped with 16 lamps LZC-420 with a maximum centered at 420 nm, while the others were kept for 1 h in the dark. Afterward, the cells were washed, trypsinized, and collected by centrifugation. The cell pellet was thoroughly washed with ice-cold PBS, and DNA was isolated using DNAzol (DNAzol®, MRC) supplemented with RNase A (100 μg/mL) according to the manufacturer's protocol. Pt content and concentration of DNA in the samples were determined by ICP-MS (Agilent Technologies, CA, USA) and UV-vis spectroscopy, respectively.

Effect of DNA repair on the toxic effects in cells. CHO-K1 and MMC-2 cells were seeded on 96-well tissue culture plates at 3,000 cells/well density in 100 μL of complete DMEM medium and cultured overnight in a humidified incubator. The medium was then removed, the tested compounds diluted in EBSS (EBSS = Earle's Balanced Salt Solution) were added to the cells, and these were then incubated for 60 min in the dark. Control cells were incubated with complex-free EBSS containing the same concentration of DMSO (less than 0.2%) as in the cells treated with Pt complexes. It was verified that this concentration of DMSO in vehicle controls did not affect the viability of the cells. After 1 h of incubation, the cells were irradiated or sham irradiated for an additional 1 h using an LZC-4 photoreactor with a blue light (maximum centered at 420 nm). After the irradiation period, the EBSS with Pt complexes was removed, and cells were cultured for 72 h in a drug-free complete DMEM medium. The number of cells in each well was determined using a standard MTT assay. The IC_{50} values were obtained from dose-response curves.

DFT Calculations. Electronic structure calculations were carried out using the Gaussian16 software package[7] at the density functional theory (DFT) level. The employed computational protocol was chosen based on the outcomes of a benchmark carried out to check the performance of the most commonly used exchange-correlation (xc) functionals to properly

reproduce the experimental UV-vis spectra in water of the two isomers. The comparison between the experimental value of λ_{max} and the corresponding computational counterparts calculated adopting several xc functionals can be found in Figure 1d. The hybrid B3LYP functional coupled with the D3 Grimme's dispersion correction for nonbonding interactions, which is able to well reproduce the electronic spectrum,[8-10] was employed for all calculations, with the Stuttgart/Dresden (SDD) relativistic effective core potential and valence basis set applied for Pt atom.[11] For all other atoms, standard double-ζ basis sets, including 6-31G* were utilized. Solvent effects were considered using the Tomasi polarizable continuum model (PCM) with water as the solvent, characterized by a dielectric constant of 78.4. TDDFT was applied to investigate excited-state properties and to study all the photoactivation pathways leading to the formation of the final photoproducts. Structural optimization and vibrational analysis confirmed the nature of the stationary points, verifying minima and transition states. Zero-point energy (ZPE) corrections were also included. The intrinsic reaction coordinate (IRC) algorithm was used to ensure accurate connection between transition states and their respective minima.[12] UV-vis absorption spectra were simulated by calculating 150 vertical electronic excitations from the optimized ground-state structures using TDDFT. A preliminary benchmark on the absorption wavelength of *Cis*-**1** was conducted to select the most appropriate exchange-correlation functional, with the B3LYP functional chosen based on its agreement with experimental values, as demonstrated in the Table S4 of supporting information. The functionals used for this purpose are B3LYP-D3,[8-10] B3PW91,[10,13,14] CAM-B3LYP,[15] PBE,[16] PBE0,[17] M05,[18] M06,[19] M06L,[20] M062X,[20] M11[21]. To characterize properly the electronic transitions, the fragment-based analysis has been computed with TheoDORE (Theoretical Density, Orbital Relaxation and Exciton analysis) software.[22]

To establish the probability for a triplet state to be populated through intersystem spin crossing (ISC), spin-orbit matrix elements have been calculated using the SOC-TD-DFT approach implemented in Orca code.[23,24] SOC values have been calculated according to the following Equation:

$$SOC_{nm} = \sqrt{\sum_i \left|\langle \psi_{S_n} \middle| \hat{H}_{SO} \middle| \psi_{T_{i,m}} \rangle\right|^2}; \qquad i = x, y, z \qquad (1)$$

Where $\hat{H}_{SO}$ is the spin-orbit Hamiltonian with effective nuclear charge. Relativistic corrections have been obtained by the zeroth order regular approximation (ZORA) and its def2-SVP basis set at the ground-state optimized geometries. Accordingly, ZORA-DEF2-SVP and SARC-ZORA-SVP have been used for the main and metal atoms, respectively. The RIJCOSX approximations was introduced to speed up calculations and very tight SCF convergence and a very large grid (Lebedev 770 points) have been set for such calculations. To identify and characterize accurately the potential triplet states involved in the photoactivation of cis and trans platinum complexes, the geometries of the triplet states identified within the vertical

approximation, located below the bright state, were initially optimized at the TD-B3LYP level. Subsequently, further optimization was performed using the unrestricted Kohn–Sham (UKS) formalism. These geometries were used as the starting point for the final UKS optimizations.[25,26]

Potential formation of Pt(II) species upon irradiation based on calculations. The effect of the intercepted excited states in the photocytotoxicity of the two investigated isomers was explored in depth by TDDFT calculations (Figure S14). *Cis*-**1** as the triplet state *Cis*-T_a can form photoproduct $[Pt^{III}(MNZ)_2(OH)_2(N_3)]$ by releasing an azidyl radical, while the *cis*-$[Pt^{II}(MNZ)(OH)_2(N_3)]^-$ species is formed from the *cis*-T_b triplet as a consequence of the photodissociation of metronidazole and azide ligands (Figure S14a). The calculated photoprodudcts match well with those detected experimentally. Starting from the adduct $^3\{[Pt^{II}(MNZ)(OH)_2N_3]^-\_N_3^{\bullet}\_\ MNZ^{+\bullet}\}$ in the triplet state, one of the hydroxide ligands of the Pt complex can be substituted by azidyl radical leading to the formation of $^3\{[Pt^{II}(MNZ)(OH)(N_3)_2]^-\_OH^{\bullet}\_\ MNZ^{+\bullet}\}$ that is calculated to be endergonic by 8.0 kcal mol^{-1}. The height of the energy barrier that is necessary to overcome is 25.3 kcal mol^{-1}. The formed complex undergoes a further substitution of one of the azide ligands by water in the next step. The process, endergonic by 1.1 kcal mol^{-1}, requires that a barrier of 22.1 kcal mol^{-1} is overcome and leads to the formation of the $^3\{[Pt^{II}(MNZ)(OH)(H_2O)(N_3)]\_OH^{\bullet}\_\ MNZ^{+\bullet} + N_3^-\}$ photoproduct in its triplet state. Finally, the displacement of the remaining azide ligand coordinated to Pt by a second water molecule completes the process, yielding the experimentally observed photoproduct $[Pt^{II}(MNZ)(OH)(H_2O)_2]^+$. This final step occurs by overcoming an energy barrier of 17.9 kcal mol^{-1} associated with the concerted transition state, leading to the endergonic formation of the product, which is 4.7 kcal mol^{-1} higher in energy respect to $^3[Pt^{II}(MNZ)(OH)(N_3)(H_2O)]$.

From triplet state *Trans*-T_a, a pathway that leads to the formation of the second experimentally observed photoproduct, the reduced *trans*-$[Pt^{II}(MNZ)_2(H_2O)(N_3)]^+$ is proposed (Figure S14b). By analogy with the mechanism operative for *Cis*-**1**, the initial step involves the substitution of one hydroxide ligand coordinated to the metal with an azidyl radical. This leads to the formation of the $^3\{[Pt^{II}(MNZ)_2(OH)(N_3)]\_N_3^{\bullet}\_\ OH^{\bullet}\}$ adduct, which is calculated to be endergonic by 8.0 kcal mol^{-1}. The substitution reaction takes place by overcoming an energy barrier of 8.8 kcal mol^{-1}. In the subsequent step, the second azidyl radical replaces the remaining hydroxyl radical coordinated to platinum, surmounting an energy barrier of 8.1 kcal mol^{-1}. As a result, the intermediate *trans*-$^3\{Pt^{II}(MNZ)_2(N_3)_2\_2OH\}$ is formed having a relative energy of 4.0 kcal mol^{-1}. The rate-determining step of the overall process is the hydrolysis of $^3[Pt^{II}(MNZ)_2(N_3)_2]$, occurring along a singlet multiplicity pathway, which requires 26.2 kcal mol^{-1} to occur. The

entire reaction is endergonic by 10.7 kcal mol^{-1}.

Table S1. Crystal data and structure refinement for *Cis*-**1** and *Trans*-**1**·2MeOH.

Compound	*Cis*-**1**	*Trans*-**1**·2MeOH
CCDC code	2373338	2373339
Empirical formula	$C_{12}H_{20}N_{12}O_8Pt$	$C_{16}H_{36}N_{12}O_{12}Pt$
Formula weight	655.49	783.66
Temperature/K	150(2)	150(2)
Crystal system	triclinic	monoclinic
Space group	P-1	I2/a
a/Å	8.83716(14)	17.3893(4)
b/Å	10.12517(17)	9.22880(10)
c/Å	11.8398(2)	18.0535(13)
α/°	96.7450(14)	90
β/°	103.1575(14)	103.922(5)
γ/°	92.8267(14)	90
Volume/Å^3	1021.27(3)	2812.2(2)
Z	2	4
ρ_{calc}mg/mm^3	2.132	1.851
μ/mm^{-1}	13.513	10.040
F(000)	636.0	1560.0
Crystal size/mm^3	0.06 × 0.04 × 0.04	0.416 × 0.271 × 0.253
Radiation	Cu Kα (λ = 1.54184)	Cu Kα (λ = 1.54184)
2Θ range for data collection	7.736 to 147.578	10.096 to 147.25
Index ranges	-10 ≤ h ≤ 10, -12 ≤ k ≤ 12, -14 ≤ l ≤ 14	-21 ≤ h ≤ 17, -11 ≤ k ≤ 11, -22 ≤ l ≤ 22
Reflections collected	29223	34306
Independent reflections	4087 [R_{int} = 0.0397, R_{sigma} = 0.0218]	2853 [R_{int} = 0.0322, R_{sigma} = 0.0110]
Data/restraints/parameters	4087/91/369	2853/0/191
Goodness-of-fit on F^2	1.071	1.170
Final R indexes [I>=2σ (I)]	R_1 = 0.0205, wR_2 = 0.0541	R_1 = 0.0167, wR_2 = 0.0420
Final R indexes [all data]	R_1 = 0.0213, wR_2 = 0.0547	R_1 = 0.0168, wR_2 = 0.0420
Largest diff. peak/hole / e Å^{-3}	0.71/-1.52	0.95/-1.11

Table S2. Selected bond lengths (Å) and bond angles (˚) for *Cis*-**1** and *Trans*-**1**·2MeOH.

Cis-**1**		*Trans*-**1**·2MeOH	
Pt1–O2	2.004(2)	Pt1–O1	2.0055(13)
Pt1–O3	2.011(3)	N1–Pt1	2.0256(16)
Pt1–N6	2.056(3)	Pt1–N10	2.0508(16)
Pt1–N14	2.059(3)	O1–Pt1–O1[1]	180.0
Pt1–N17	2.041(3)	N1–Pt1–N10	92.89(7)
Pt1–N20	2.034(3)	N10–Pt1–N10[1]	180.0
O2–Pt1–O3	176.73(9)	N1–Pt1–N1[1]	180.0
N6–Pt1–N14	88.19(11)		
N20–Pt1–N17	91.57(12)		
N17–Pt1–N14	92.05(11)		
N20–Pt1–N6	88.17(11)		

[1]1/2-X,1/2-Y,3/2-Z

Table S3. Hydrogen bonds for *Cis*-**1** and *Trans*-**1**·2MeOH.

**Crystal**	**D**	**H**	**A**	**d(D-H)/Å**	**d(H-A)/Å**	**d(D-A)/Å**	**D-H-A/°**
Cis-**1**	O1	H1	O2[1]	0.849(8)	1.836(15)	2.672(3)	168(6)
	O2	H2	N20[1]	0.847(8)	2.434(14)	3.266(4)	168(5)
	O3	H3	O16B[2]	0.844(8)	2.15(3)	2.922(7)	152(5)
	O9A	H9A	O1[3]	0.849(8)	2.00(6)	2.772(7)	151(12)
Trans-	O1	H1	O200[4]	0.84	1.95	2.780(2)	168.6
**1**·2MeOH	O9	H9	O100[5]	0.84	1.88	2.693(2)	162.8
	O100	H100	O1[6]	0.84	1.82	2.657(2)	174.3
	O200	H200	O9	0.84	1.94	2.736(2)	157.4

[1]1-X,-Y,1-Z; [2]1+X,+Y,+Z; [3]-1+X,+Y,-1+Z; [4]3/2-X,-1/2+Y,3/2-Z; [5]-1/2+X,1/2-Y,-1/2+Z; [6]1-X,1/2+Y,3/2-Z

Table S4. TDDFT benchmark for the reproduction of the experimental spectrum of *Cis*-**1** on the structure optimized at B3LYP-D3/6-31G* level in H_2O implicit solvent, λ_{exp} = 290 nm.

Functional	State[a]	λ	ΔE	*f*	MO Contribution[b]
**B3LYP-D3**	S1	456	2.72	0.0011	H→L+2 96%
	S28	286	4.32	0.2247	H-7→L+1 26%, H-9→L 17%
**CAMB3LYP**	S1	418	2.97	0.0011	H→L+2 88%
	S12	277	4.48	0.5927	H-3→L+1 33%, H-2→L 32%
**PBE**	S1	636	1.95	0.0032	H→L 99%
	S45	311	3.98	0.1692	H-8→L 14%, H-7→L+1 19%
**PBE0**	S1	447	2.77	0.0011	H→L+2 91%
	S26	279	4.45	0.1662	H-6→L 20%, H-6→L+2 19%
**M05**	S1	477	2.60	0.0008	H→L+2 94%
	S20	287	4.32	0.1420	H-16→L 26%, H-16→L+1 22%, H-6→L 14%
**M06**	S1	474	2.62	0.0009	H→L+2 93%
	S22	296	4.19	0.1603	H-5→L 26%, H-4→L+1 28%
**M052X**	S1	434	2.85	0.0008	H-1→L+2 57%, H→L+3 26%
	S14	267	4.63	0.6442	H-3→L 36%, H-2→L+1 32%
**M062X**	S1	443	2.80	0.0007	H→L+2 82%
	S14	268	4.62	0.6843	H-3→L 23%, H-3→L+1 18%, H-2→L 21%, H-2→L+1 19%

[a]Both bright state (S1) and singlet state, where the most important bands are centered, are reported. [b]Only contributions larger than 14% are reported.

Table S5: B3LYP-D3 excitation energies (ΔE, eV), absorption wavelength (λ, nm), oscillator strength (*f*), MO contribution (%) for selected transitions Tr for *Cis*-**1** and *Trans*-**1**.

Comp.	Tr[a]	ΔE	λ	*f*	MO contributions [b]	Theoretical Assignment
Cis-**1**	Tr1	2.72	456	0.001	H→L+2 96%	21 % LC (N_3) / 31% LMCT (OH/N_3 to Pt) / 27% LLCT (OH/N_3 to MNZ)
	Tr2	3.61	344	0.036	H-2→L 84%	22% MLCT (Pt to MNZ) / 74% LLCT (OH/N_3 to MNZ)
	Tr3	3.66	338	0.032	H-3→L 73%, H-2→L+1 16%	74% LLCT (OH/N_3 to MNZ)
	Tr4	3.98	312	0.077	H-12→L 16%, H-4→L 69%	73% LC (MNZ) / 25% LLCT (OH to MNZ)
	Tr5	4.05	306	0.032	H-6→L 13%, H-4→L+1 59%	59% LC (MNZ) / 38% LLCT (OH to MNZ)
	Tr6	4.16	298	0.066	H-5→L+2 56%	30% LMCT (OH/MNZ to Pt) / 42% LLCT (OH/MNZ to N_3)
	Tr7	4.22	294	0.152	H-6→L 43%, H-5→L 10%, H-5→L+1 17%	30% LC (MNZ) / 62% LLCT (N_3 to MNZ)
	Tr8	4.24	293	0.064	H-6→L+1 27%, H-5→L+1 29%, H-5→L+2 16%	39% LC (MNZ / 48% LLCT (N_3 to MNZ)
	Tr9	4.29	289	0.065	H-6→L+2 64%	31% LMCT (MNZ to Pt) / 47% LLCT (MNZ to N_3)
	Tr10	4.34	286	0.225	H-9→L 17%, H-8→L 14%, H-7→L+1 26%, H-6→L+1 12%	53% LC (MNZ) / 39% LLCT (MNZ/OH/N_3 to MNZ)
	Tr11	4.36	285	0.100	H-9→L 22%, H-8→L 28%	44% LC (MNZ) / 47% LLCT (MNZ/OH/N_3 to MNZ)
	Tr12	4.86	255	0.071	H-8→L+3 33%, H-7→L+3 34%	37% LMCT (MNZ/ N_3 to Pt) / 35% LLCT (MNZ/ N_3 to OH)
Trans-**1**	Tr1	3.04	408	0.006	H→L+1 98%	86% LLCT (N_3 to MNZ)
	Tr2	3.34	371	0.030	H-1→L+1 99%	28% MLCT (Pt to MNZ) / 70% LLCT (OH to MNZ)

Tr3	3.98	312	0.175	H-6→L+1 11%,<br>H-5→L+1 32%,<br>H-4→L 49%	94% LC (MNZ)
Tr4	4.11	302	0.225	H-3→L+2 47%,<br>H-4→L+2 24%,<br>H-3→L+3 13%	28% LC (N_3)/<br>41% LMCT (MNZ to Pt)
Tr5	4.20	295	0.062	H-2→L+3 72%	28%LC (OH) /<br>49% LMCT (N_3 to Pt)
Tr6	4.27	291	0.405	H-6→L+1 47%,<br>H-7→L 30%	74% LC (MNZ) /<br>19% LLCT (OH to MNZ)
Tr7	4.41	281	0.148	H-3→L+3 68%	43% LC (N_3/OH) /<br>42% LMCT (N_3 to Pt)

Tr = transition number. [a]Only vertical transitions with oscillator strength greater than 0.030 are reported, with the exception of the vertical transition in the 400−500 nm range with oscillator strength greater than 0.0001. [b]Only contributions larger than 10% are reported. MNZ = metronidazole.

Table S6. Natural Transition Orbitals (NTOs) for the most important transitions of *Cis*-**1**.

	Transitions			
	Tr1 (456nm)	Tr2 (344nm)	Tr3 (338nm)	Tr4 (312nm)
**Acceptor**	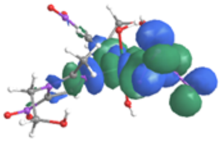	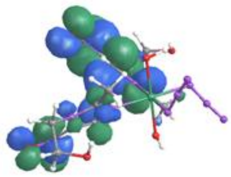	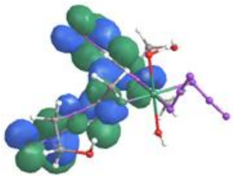	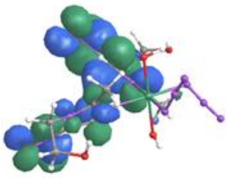
**Donor**	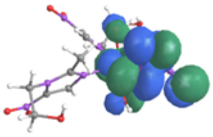	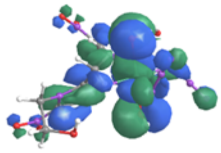	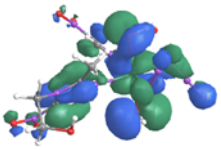	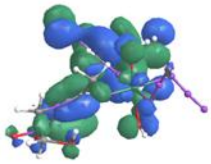
	Tr5 (306nm)	Tr6 (298nm)	Tr7 (294nm)	Tr8 (293nm)
**Acceptor**	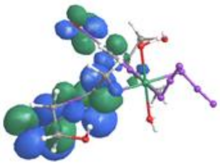	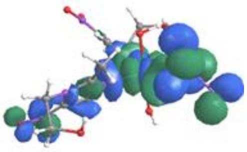	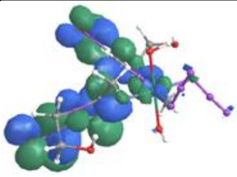	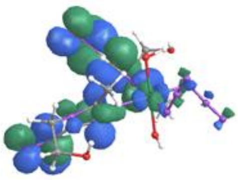
**Donor**	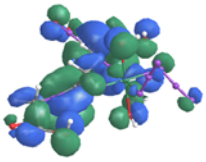	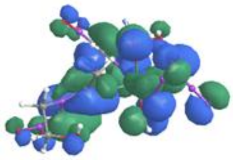	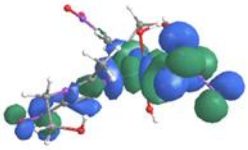	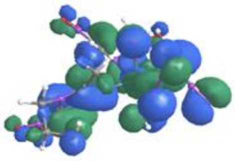
	Tr9 (289 nm)	Tr10 (286 nm)	Tr11(285 nm)	Tr12(255 nm)
**Acceptor**	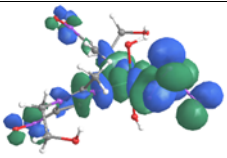	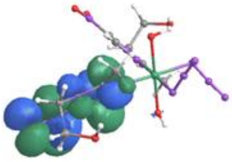	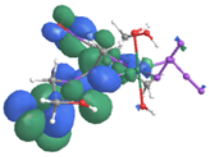	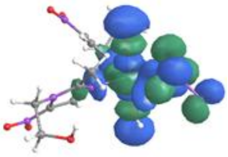
**Donor**	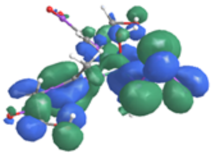	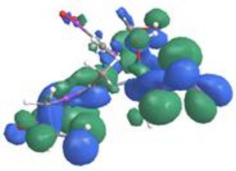	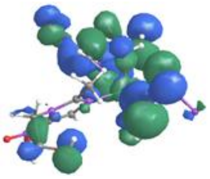	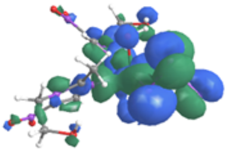

Table S7. NTOs for the most important transitions of *Trans*-**1**.

	Transitions			
	Tr1 (408 nm)	Tr2 (371 nm)	Tr3 (312 nm)	Tr4 (302 nm)
**Acceptor**	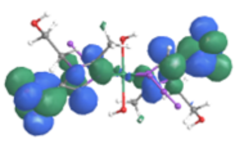	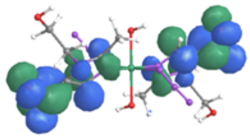	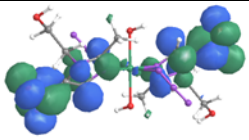	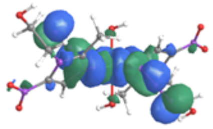
**Donor**	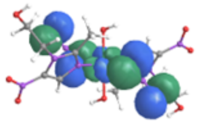	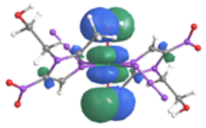	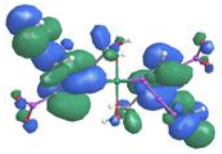	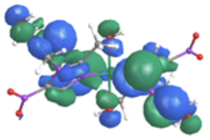
	Tr5 (295 nm)	Tr6 (291 nm)	Tr7 (281 nm)	
**Acceptor**	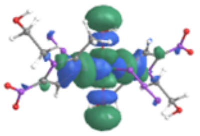	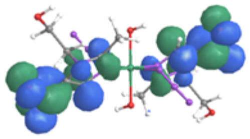	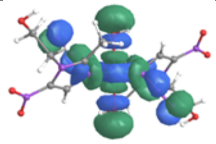	
**Donor**	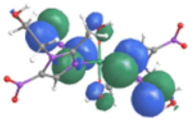	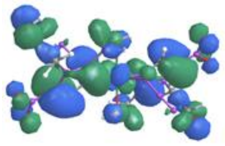	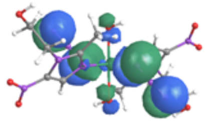	

Table S8. Cyclic voltammogram data for complexes and corresponding ligands.

Compound	Pt^{IV}/Pt^{II}	$Ar\text{-}NO_2/Ar\text{-}NO_2^{\cdot -}$	
	E_{pc}	E_{pa}	E_{pc}
Cis-**1**	-1.27 V	-1.45 V	-1.60 V
Trans-**1**	-1.28 V	-1.42 V	-1.61 V
Metronidazole		-1.52 V	-1.61 V

[a]E_{pa} and E_{pc} are the anodic and the cathodic peak potentials, respectively. Scan rate = 100 mV s^{-1}.

Table S9. Observed and calculated masses of photoproducts of *Cis*-**1** (50 μM) after 1 h irradiation (463 nm) determined by LC-MS (mobile phase: water and acetonitrile). See Figure **S12a** and **c**.

Peak	Retention time/min	Mass calculated	Mass observed	Formula
c1	3.6	475.04	474.93	$[Pt^{II}(MNZ)(HCOOH)_2(HO)]^+$
c2	5.8	172.07	171.92	{metronidazole + H^+}

Table S10. Observed and calculated masses of photoproducts of *Tras*-**1** (50 μM) after 1 h irradiation (463 nm) determined by LC-MS (mobile phase: water and acetonitrile). See Figure **S12b** and **d**.

Peak	Retention time/min	Mass calculated	Mass observed	Formula
t1	3.3	627.07	627.05	$[Pt^{III}(MNZ)_2(HCOO)_2]^+$
t2	4.3	571.10	571.05	$[Pt^{III}(MNZ)_2(OH)_2]^+$
t3	7.2	624.10	624.09	$[Pt^{III}(MNZ)_2(HCOO)(N_3)]^+$
t4	10.1	620.13	620.10	$[Pt^{II}(MNZ)_2(CH_3CN)(N_3)]^+$
t5	16.8	594.09	594.12	$\{[Pt^{II}(MNZ)_2(OH)_2] + Na\}^+$

Table S11. B3LYP-D3 excitation energies (ΔE, eV), MO contribution (%) and theoretical assignment for the singlet states of *Cis*-**1** and *Trans*-**1** involved in the ISC process, calculated by B3LYP functional.

Comp.	Tr[a]	ΔE	λ	*f*	MO contributions [b]	Theoretical Assignment
Cis-**1**	S1	2.72	456	0.0011	H→L+2 96%	21 % LC (N_3) / 31% LMCT (OH/N_3 to Pt) / 27% LLCT (OH/N_3 to MNZ)
Trans-**1**	S1	2.55	485	0.0000	H→L+2 97%	44 % LC (N_3) / 30% LMCT (N_3 to Pt)
	S2	2.94	422	0.0000	H-1→L+2 97%	31 % LC (OH) / 27% LMCT (OH to Pt) 20% MLCT (Pt to N_3)
	S3	3.03	409	0.0000	H→L 97%	86% LLCT (N_3 to MNZ)
	S4	3.04	408	0.0063	H→L+1 98%	86% LLCT (N_3 to MNZ)

Tr = transition number. MNZ = metronidazole.

Table S12. B3LYP-D3 excitation energies (ΔE, eV), MO contribution (%) and theoretical assignment for the triplet states of *Cis*-**1** and *Trans*-**1** located below the bright state calculated by B3LYP functional.

Comp.	Tr[a]	ΔE	λ	MO contributions [b]	Theoretical Assignment
Cis-**1**	T1	2.39	519	H→L+2 93%	29% LMCT (OH to Pt) / 24% LC (N_3)
	T2	2.71	458	H-1→L+2 75%, H→L+3 20%	32% MC / 32% LMCT (OH/ N_3 to Pt) / 23% LLCT (OH/ N_3 to MNZ)
Trans-**1**	T1	2.22	560	H→L+2 97%	45% LC (N_3) / 29% LMCT (OH to Pt)
	T2	2.66	466	H-1→L+2 95%	30% LC(OH) / 23% MLCT Pt to N_3) / 24% LMCT (OH to Pt)
	T3	2.73	454	H-3→L+2 81%	57% LC(OH) / 33% LMCT (N_3 to Pt)
	T4	2.83	438	H-2→L+2 55%	67% LC(OH) / 24% LMCT (N_3 to Pt)
	T5	2.84	437	H-5→L 27%, H-4→L+1 26%	93% LC (MNZ)
	T6	2.84	437	H-2→L+2 24%, H-5→L+1 20%, H-4→L 19%	84% LC (MNZ)
	T7	2.91	425	H-1→L+3 94%	32% MLCT (Pt to MNZ) / 19% LC (OH)

Tr = transition number. MNZ = metronidazole.

Table S13. NTOs for the singlet state (bright state) of *Cis*-**1**.

	Transitions
	S1 (456 nm)
**Acceptor**	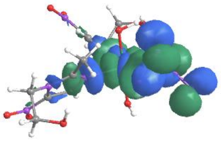
**Donor**	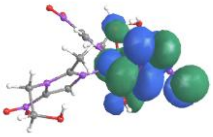

Table S14. NTOs for the singlet states below the bright state of *Trans*-**1**.

	Transitions			
	S1 (485nm)	S2 (422nm)	S3 (409 nm)	S4 (408 nm)
**Acceptor**	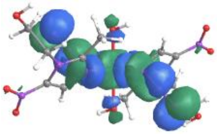	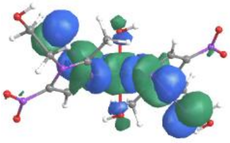	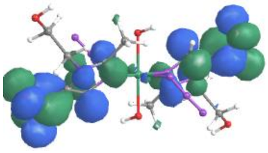	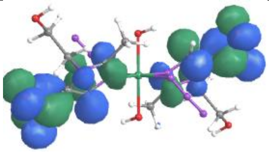
**Donor**	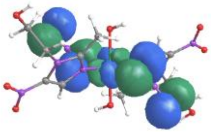	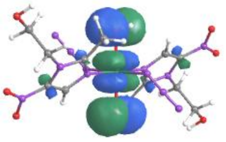	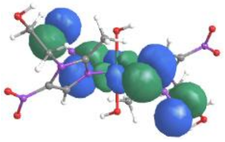	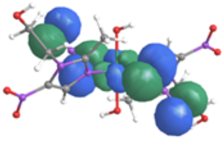

Table S15. NTOs for the triplet states of *Cis*-**1**.

	Transitions	
	T1 (519 nm)	T2 (458nm)
**Acceptor**	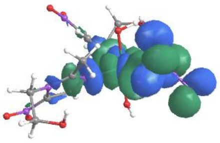	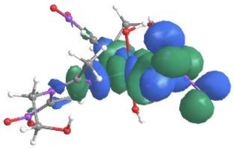
**Donor**	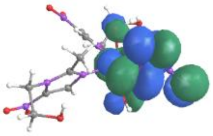	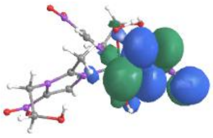

Table S16. NTOs for the triplet states of *Trans*-**1**.

	Transitions			
	T1 (560 nm)	T2 (466 nm)	T3(454 nm)	T4(438 nm)
**Acceptor**	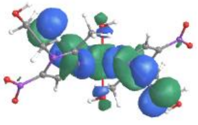	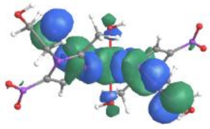	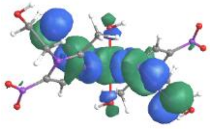	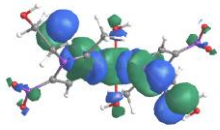
**Donor**	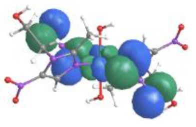	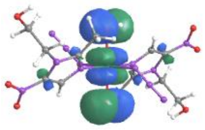	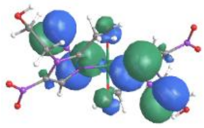	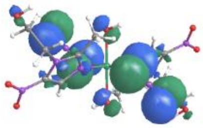
	T5 (437 nm)	T6 (437 nm)	T7(425 nm)	
**Acceptor**	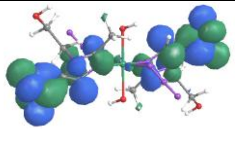	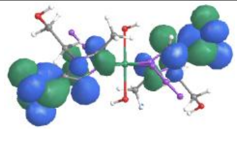	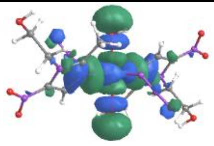	
**Donor**	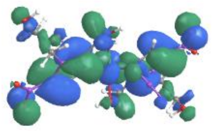	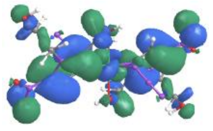	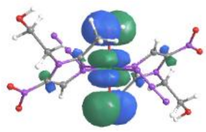	

Table S17. Spin orbit coupling constants (SOC, cm^{-1}) between the bright state and triplet states lying below, together with their adiabatic energy difference (ΔE, eV) in parentheses, calculated for *Cis*-**1** and *Trans*-**1**.

$S_n \rightarrow T_m$	**Sn**	**Tm**						
		1	2	3	4	5	6	7
Cis-**1**	1	56.07 (0.33)	577.81 (0.01)					
Trans-**1**	1	42.77 (0.34)						
	2		39.41 (0.28)	91.65 (0.21)	216.00 (0.11)	334.01 (0.10)	17302 (0.10)	
	3		11.36 (0.37)	2.49 (0.30)	84.19 (0.20)	33.00 (0.20)	391.10 (0.19)	222.79 (0.12)
	4		20.07 (0.38)	60.29 (0.31)	16.45 (0.21)	145.15 (0.20)	21.30 (0.20)	83.02 (0.12)

Table S18. Most relevant bond lengths (Å) in *Cis*-T_a, *Cis*-T_b and *Trans*-T_a triplet structures together with spin density values (SD in a.u.) on the platinum metal center and the most important atoms of the coordinated ligands.

		Cis-**1**		*Trans*-**1**
		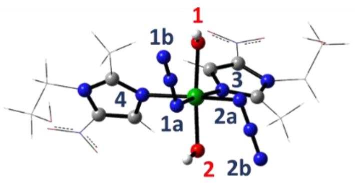		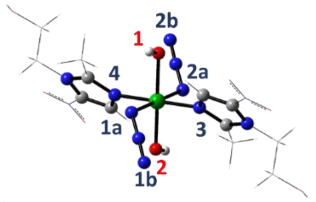
		Cis-T_a	*Cis*-T_b	*Trans*-T_a
Bond length (Å)	Pt-N_{1a}	2.059	2.057	2.569
	Pt-N_{2a}	3.558	2.611	2.591
	Pt-N_3	2.090	2.095	2.060
	Pt-N_4	2.273	3.644	2.071
	Pt-OH_1	2.018	1.942	1.999
	Pt-OH_2	2.040	1.950	1.972
Spin density values (SD in a.u.)	Pt	0.721	0.715	0.631
	N_1	0.019 (a)	0.009 (a)	0.264(a)
		0.046 (b)	0.012(b)	0.312 (b)
	N_2	0.427(a)	0.437 (a)	0.264 (a)
		0.430(b)	0.440 (b)	0.320 (b)
	N_3	0.013	0.001	0.010
	N_4	0.021	0.002	0.010
	O_1	0.050	0.249	0.213
	O_2	0.226	0.295	0.140

Table S19. IC_{50} values and photocytotoxic indices (PI) for complexes in A2780 and cisplatin-resistant A2780cis ovarian cancer cells after 1 h incubation, 1 h irradiation (blue 465nm, green 520 nm) and 72 h further incubation under normoxia (21% O_2). CDDP (cisplatin) and complex **FM190** were studied for comparison.

Cell	Condition	**IC_{50} (μM)**[a]			
		Cis-**1**	*Trans*-**1**	**FM190**	**CDDP**
A2780	Dark	> 100	> 100	11.6±1.8	0.7±0.1
	465 nm	46.1±7.2	9.4±2.7	1.1±0.1	0.9±0.5

	520 nm		> 100	34.4±1.1	9.9±1.6	0.82±0.03
	PI	Blue	> 2.1	> 10.6	10.5	-
		Green	-	> 2.9	1.2	-
A2780cis	Dark		> 100	> 100	21.7±0.1	9.7±1.4
	465 nm		40.4±3.1	25.2±0.8	2.8±0.7	7.68±0.04
	520 nm		> 100	> 100	19.3±1.8	10.2±1.2
	PI	Blue	> 2.4	> 3.9	7.8	-
		Green	-	-	1.1	-

[a] All IC_{50} values were obtained from at least two independent experiments.

Table S20. Cellular Pt accumulation from complexes (10 μM) in SW780 bladder cancer cells after 1 h incubation, 1 h irradiation (blue 465 nm) under normoxia (21% O_2) and hypoxia (1% O_2).

Complex	Cellular Pt accumulation (ng/10^6 cells)			
	Normoxia (21% O_2)		Hypoxia (1% O_2)	
	Dark (2 h)	465 nm	Dark (2 h)	465 nm
Cis-**1**	0.44±0.04	0.43±0.13	0.45±0.03	0.88±0.08
Trans-**1**	1.36±0.27	1.17±0.19	1.06±0.19	2.44±0.47

Table S21. Cellular Pt distribution after incubation of SW780 bladder cancer cells with complexes (10 μM) for 2 h in the dark under normoxia (21% O_2).

Complex	Cellular Pt ratio (%)		
	Nuclei	Mitochondria	Cytoplasm
Cis-**1**	37.2±4.3	17.3±6.3	45.4±6.1
Trans-**1**	49.5±6.2	15.5±1.8	35.0±8.0

Table S22. Comparison of photoproducts of diazido Pt(IV) complexes detected by LC-MS.

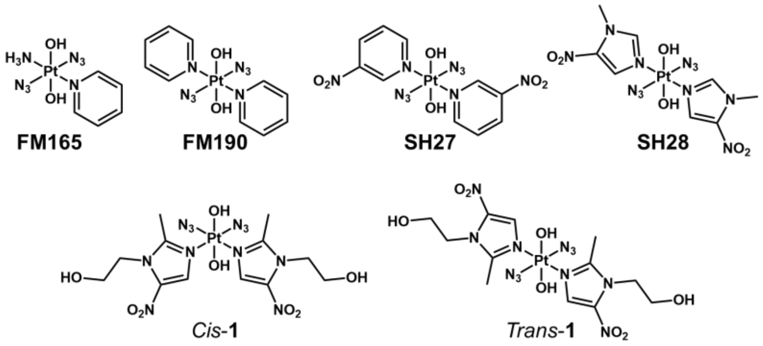

Complex		FM165[27]	FM190[28, 29]			HS27[30]	HS28[31]	*Cis*-1	*Trans*-1
**Configuration**		*t,t,t*	*t,t,t*			*t,t,t*	*t,t,t*	*c,c,t*	*t,t,t*
**Amine ligand (Am)[a]**		Am_1 = Py<br>Am_2 = NH_3	Am_1/Am_2 = Py			Am_1/Am_2 = 3-NO_2-Py	Am_1/Am_2 = 1-Me-5-NO_2-Im	Am_1/Am_2 =MNZ	
**Irradiation wavelength/nm**		365	420	463	463	463	463	463	463
**HPLC mobile phase B (+ 0.1%FA)[b]**		MeOH	MeCN	MeOH	MeCN	MeCN	MeCN	MeCN	MeCN
**Pt(II) photo-products**	$[Pt(Am_1)(Am_2)(N_3)(CH_3CN)]^+$		√			√	√		√
	$[Pt(Am)_3(N_3)]^+$						√		
	$\{[Pt(Am)_2(HCOO)_3] + H\}^+$			√					
	$\{[Pt(Am)_2(OH)_2]+ Na\}^+$								√
	$[Pt(Am)(HCOOH)_2(OH)]^+$							√	
**Pt(III)**	$[Pt(Am_1)(Am_2)(N_3)(OH)]$	√							

**photo-products**	$\{Pt(Am)_2(N_3)(HCOO)\}^+$		√						√
	$[Pt(Am_1)(Am_2)(OH)_2]^+$	√	√						√
	$[Pt(Am)_2(HCOO)_2]^+$					√			√
	$\{[Pt(Am)_2(HCOO)(OH)_2(H_2O)] + Na\}^+$			√	√				
	$[\{Pt(Am)_2(HCOO)_3\} + Na]^+$				√				
**Pt(IV) photo product**	$Pt(Am_1)(Am_2)(N_3)_3(O^-)$	√							
	$\{[Pt(Am)_2(N_3)(OH)_3] + Na\}^+$			√	√	√	√		
	$\{[Pt(Am)_2(OH)_4] + Na\}$+			√	√				
	$\{[Pt(Am)_2(OH)_3]_2(\mu\text{-}O_2) + Na\}^+$			√	√				
	$[Pt(Am)_2(HCOO)_3]^+$						√		

a Py = pyridine; 3-NO_2-Py = 3-nitropyridine; 1-Me-5-NO_2-Im = 1-methyl-5-nitroimidazole; MNZ = metronidazole. [b] HPLC mobile phase A is H_2O + 0.1%FA in all cases here.

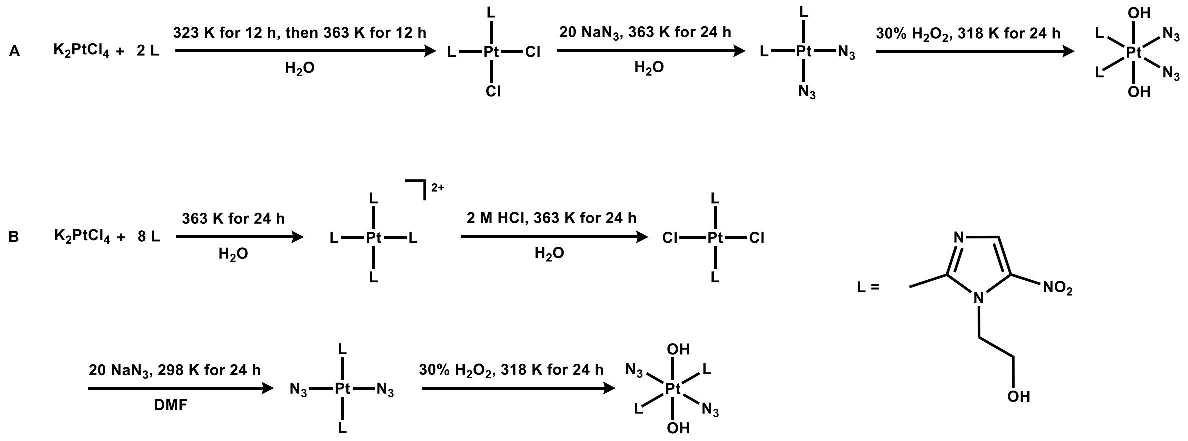

Scheme S1. The synthetic routes for Pt(IV) complexes *Cis*-**1** (A) and *Trans*-**1** (B).

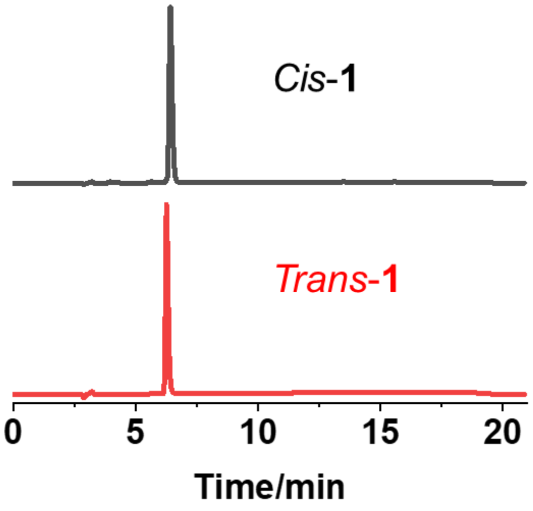

Figure S1. HPLC chromatograms for complexes *Cis*-**1** and *Trans*-**1** (detection wavelength 254 nm, Mobile phase A: H_2O+0.1% FA; B: ACN+0.1% FA, 10-30% B in 10 min, 30% B for 5 min).

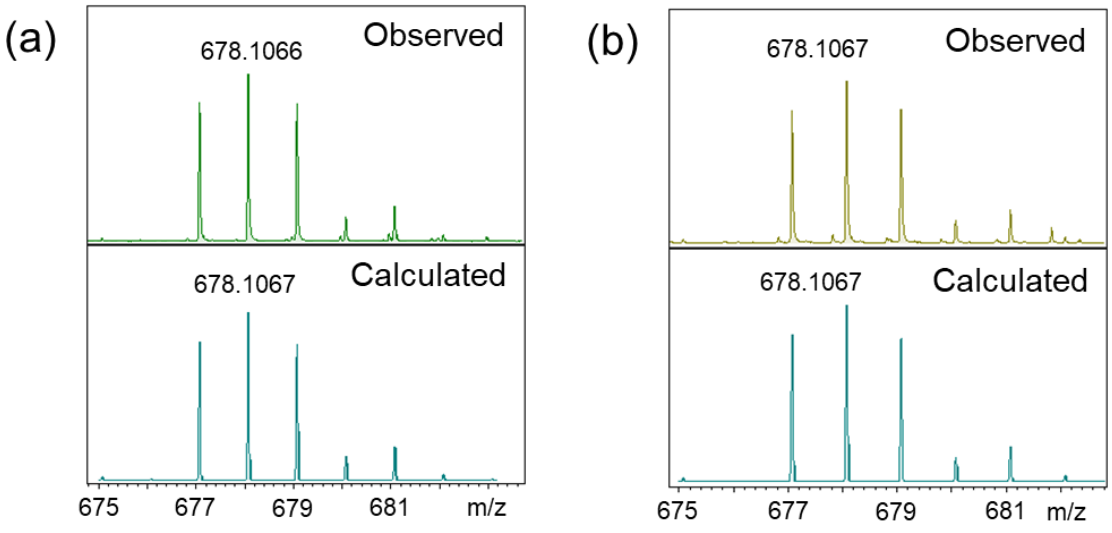

Figure S2. HR-ESI-MS of complexes (a) *Cis*-**1**and (b) *Trans*-**1** in positive mode (+Na^+).

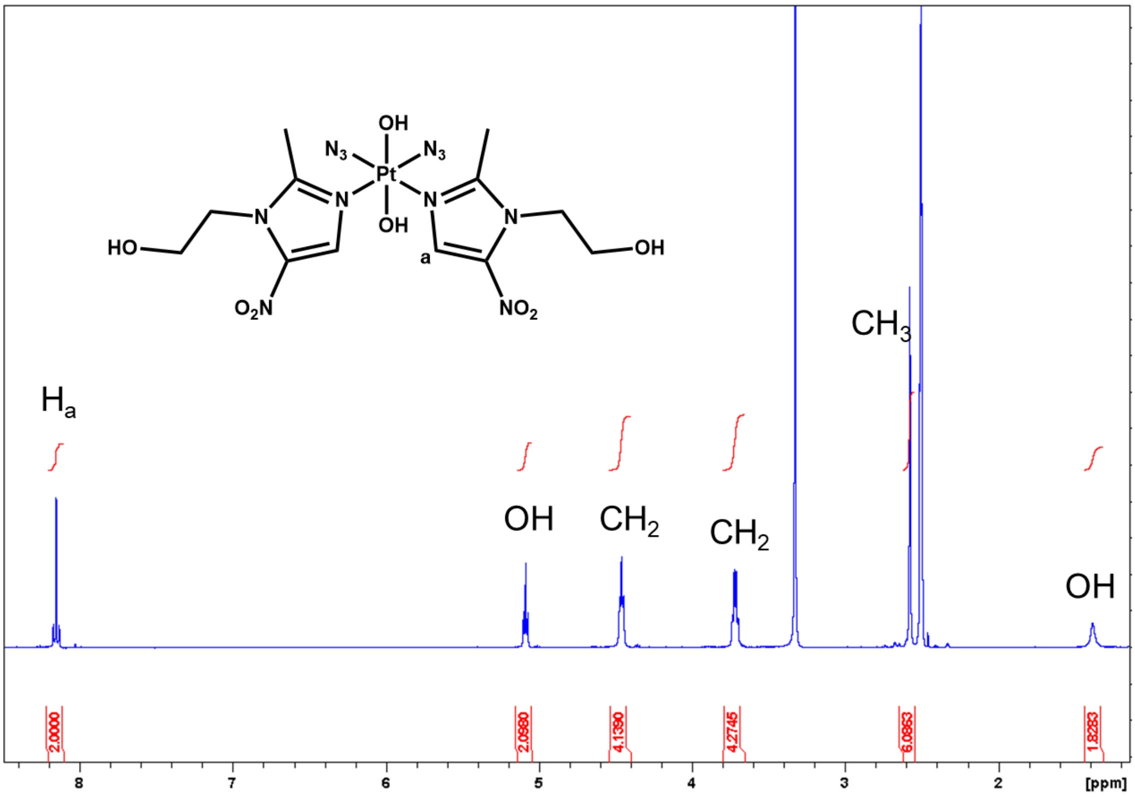

Figure S3. 500 Hz ^{1}H NMR spectrum of complex *Cis*-**1** in DMSO-d_6 at 298 K.

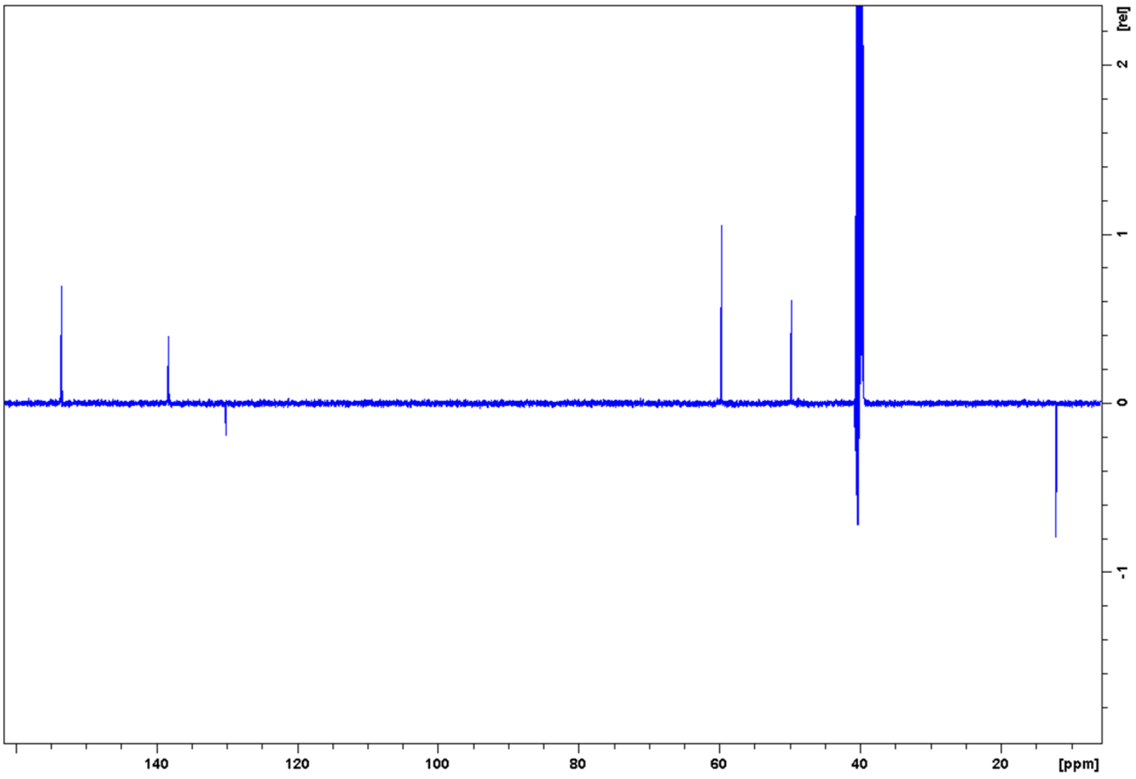

Figure S4. 125 Hz ^{13}C-{^{1}H} APT NMR spectrum of complex *Cis*-**1** in DMSO-d_6 at 298 K.

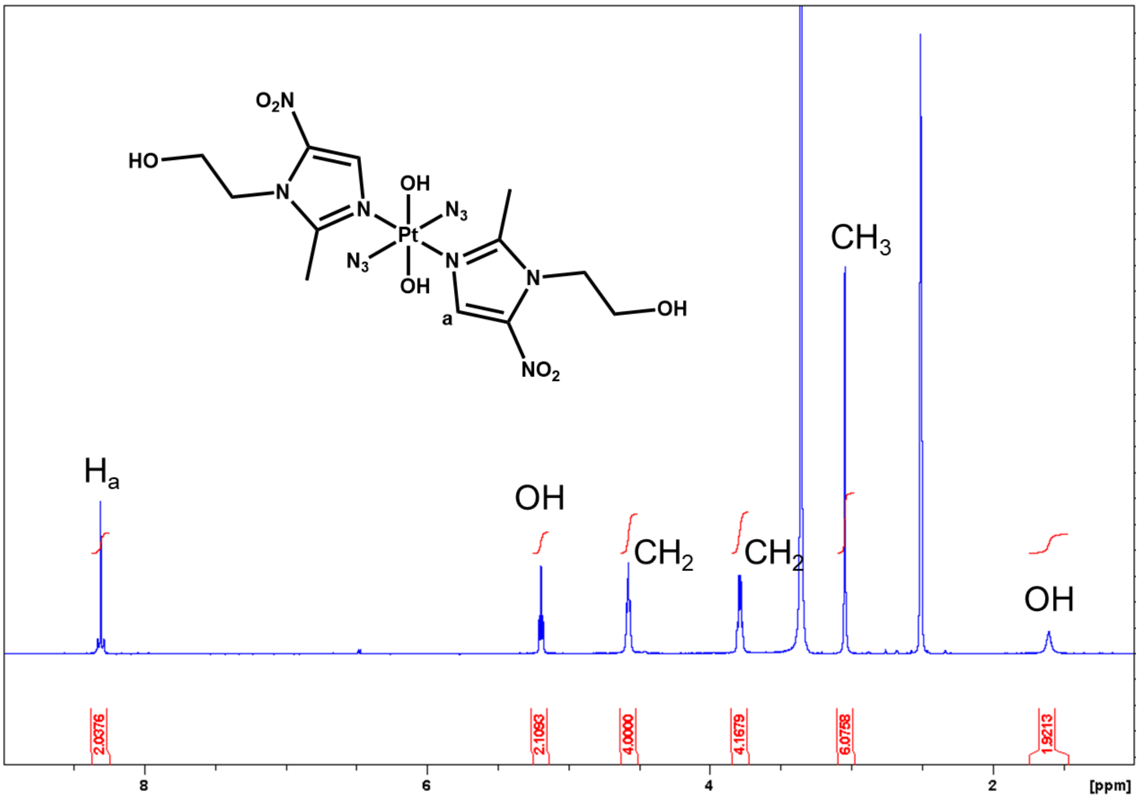

Figure S5. 500 Hz ^{1}H NMR spectrum of complex *Trans*-**1** in DMSO-d_6 at 298 K.

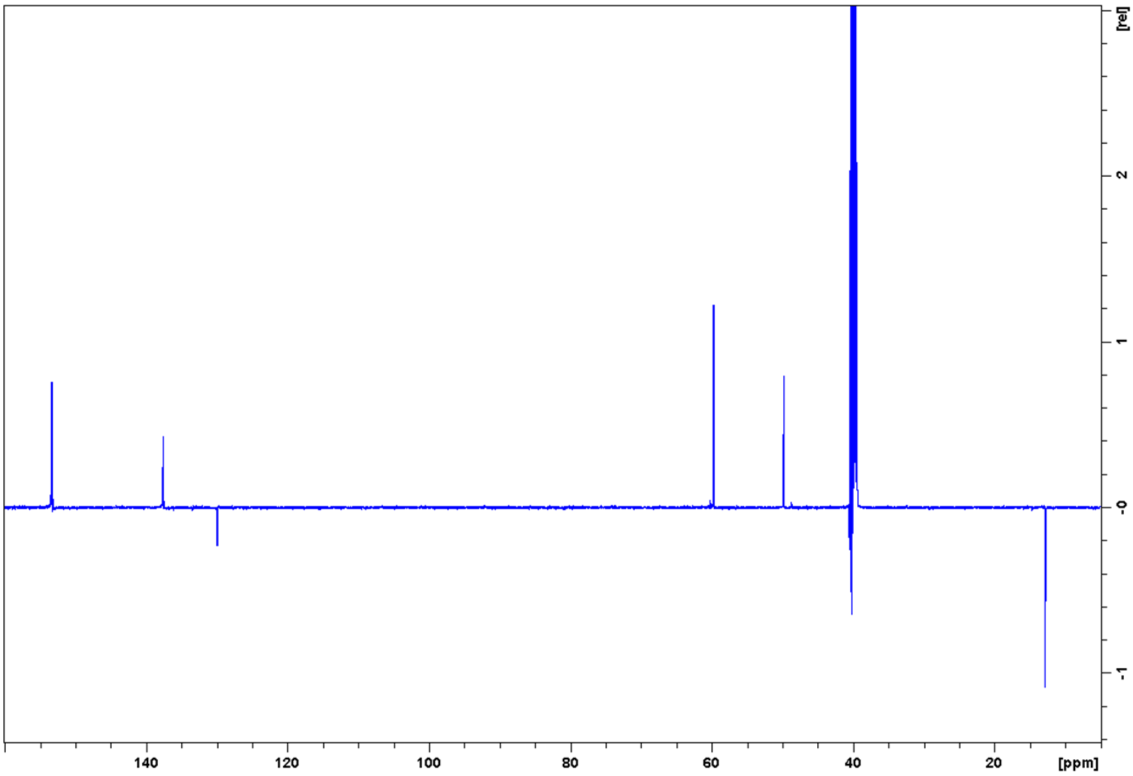

Figure S6. 125 Hz ^{13}C-{^{1}H} APT NMR spectrum of complex *Trans*-**1** in DMSO-d_6 at 298 K.

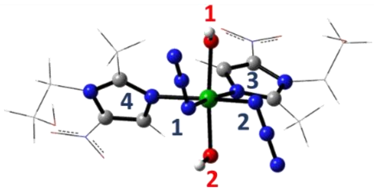

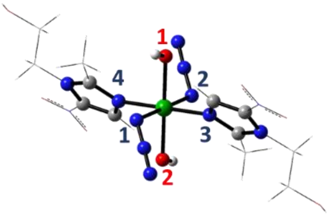

Cis-**1**		*Trans*-**1**
2.060	Pt-N_1	2.097
2.060	Pt-N_2	2.097
2.092	Pt-N_3	2.062
2.094	Pt-N_4	2.062
2.042	Pt-OH_1	2.042
2.043	Pt-OH_2	2.043

Figure S7. B3LYP-D3 optimized structure of *Cis*-**1** and *Trans*-**1** isomers along with the most relevant geometrical parameters. Bond lengths are in Å.

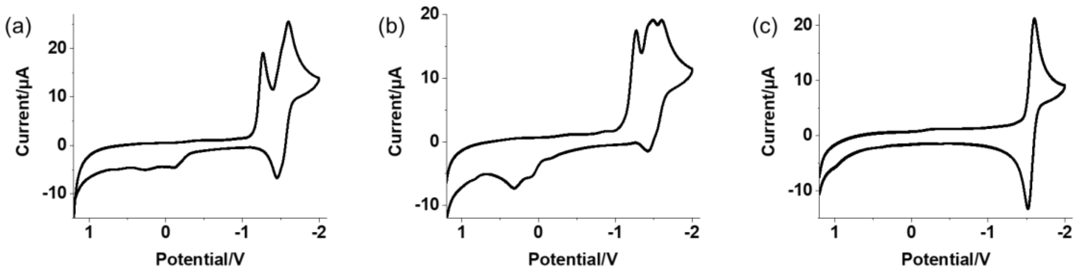

Figure S8. Cyclic voltammograms of complexes (a) *Cis*-**1**, (b) *Trans*-**1**, and (c) and metronidazole, (1 mM in 0.1 M NBu_4PF_6-DMF, under N_2).

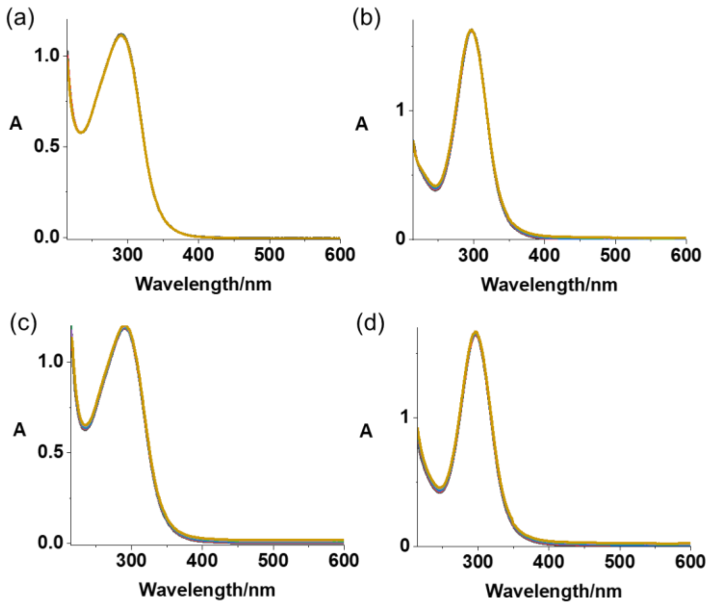

Figure S9. UV-vis spectra showing the dark stability of complexes *Cis*-**1** and *Trans*-**1** in air (a for *Cis*-**1**; b for *Trans*-**1**) and N_2-saturated (c for *Cis*-**1**; d for *Trans*-**1**) MillQ H_2O at 310 K for 2 h.

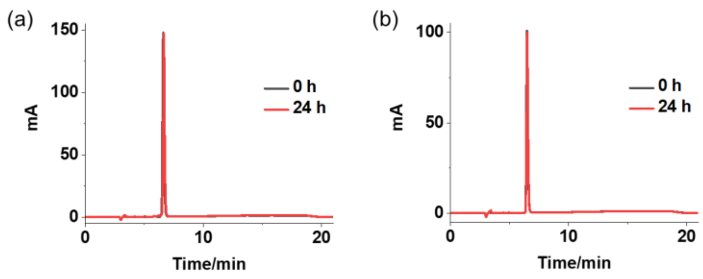

Figure S10. HPLC of (a) *Cis*-**1** and (d) *Trans*-**1**, 50 μM) in aqueous solution after incubation in the dark at 310 K for 0 and 24 h, detection at 254 nm.

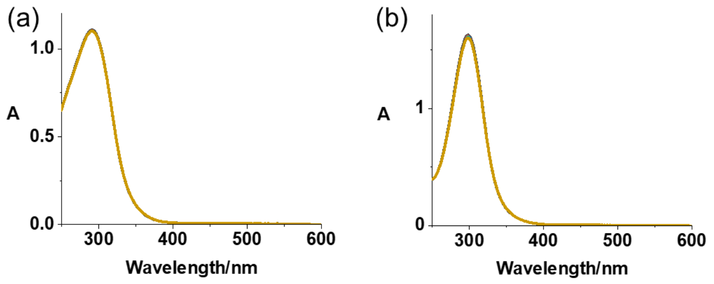

Figure S11. UV-vis spectra showing the dark stability of complexes *Cis*-**1** and *Trans*-**1** (50 µM) in the presence of 2 mM GSH in MillQ H_2O at 298 K for 2 h.

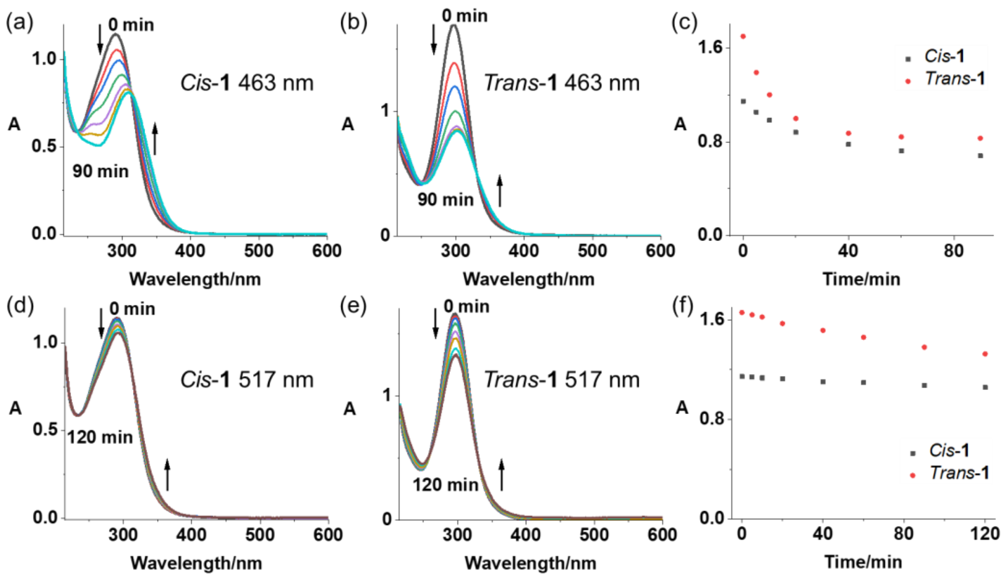

Figure S12. Time dependent UV-vis spectral changes for complexes *Cis*-**1** and *Trans*-**1** in N_2-saturated MillQ H_2O exposed to blue (463 nm, (a) *Cis*-**1**; (b) *Trans*-**1**) or green (517 nm, (d) *Cis*-**1**; (e) *Trans*-**1**) light at 298 K; (c) and (f), plots of time-dependent absorbance changes for complexes at the absorption maximum (290 nm for *Cis*-**1**; 297 nm for *Trans*-**1**) upon irradiation with light of different wavelengths ((c) 463 nm; (f) 520 nm).

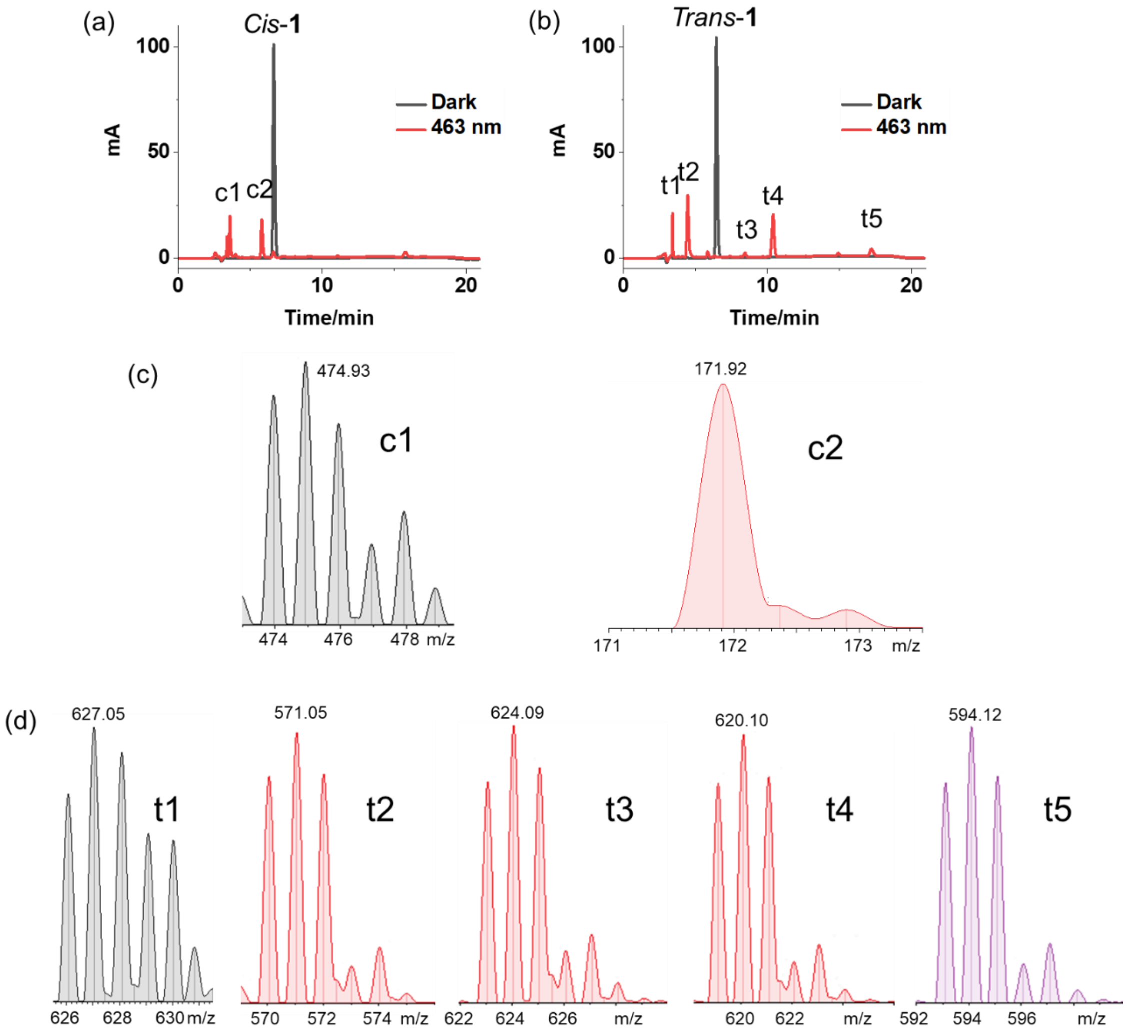

Figure S13. HPLC for 50 µM complex in water, (a) *Cis*-**1** and (b) *Trans*-**1** in the dark (black traces) and after 1 h irradiation with blue light (463 nm). Mass spectra of photoproducts (c) c1–c2 and (d) t1–t5 are presented and the possible assignments are listed in Tables **S9** and **S10**.

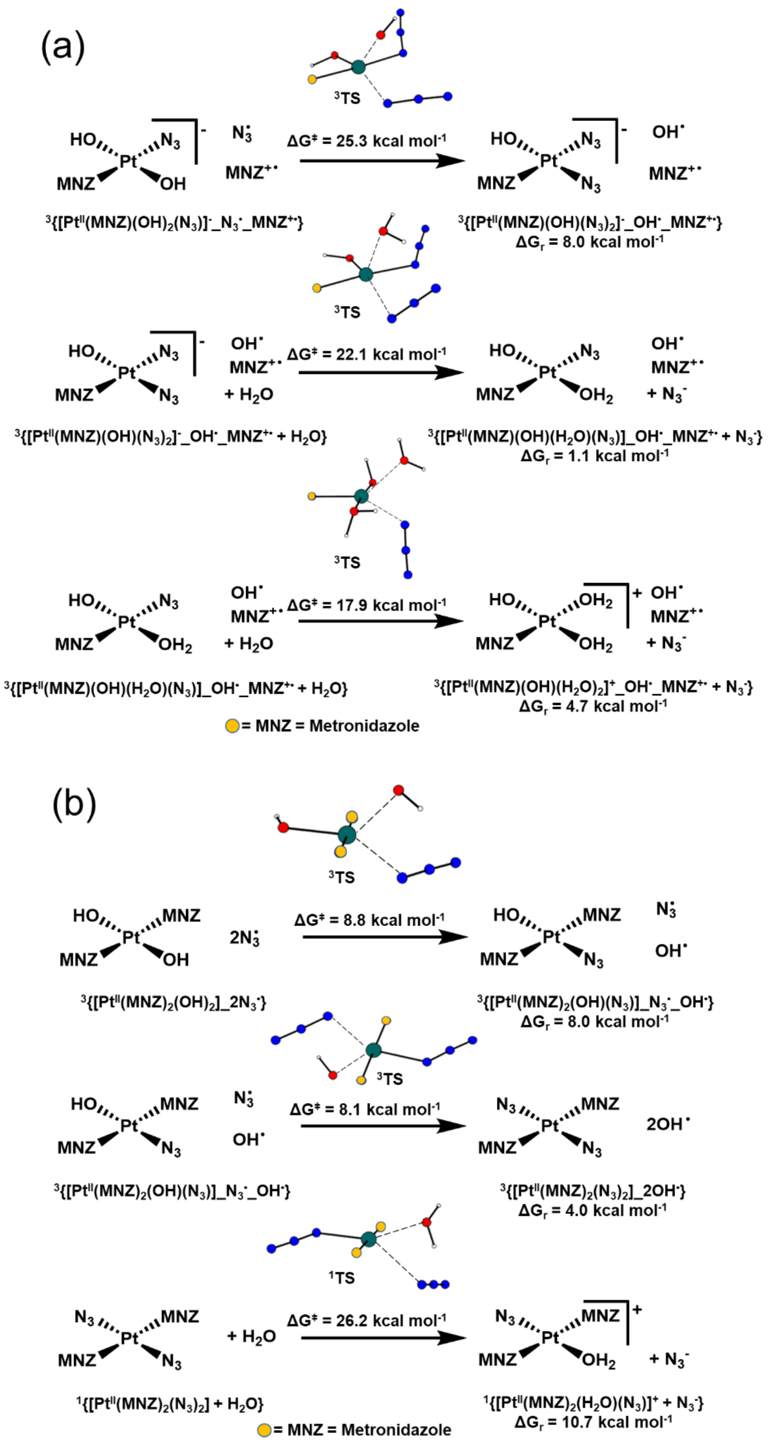

Figure S14. Proposed excited triplet state reaction mechanisms for the formation of (a) the photoproducts *cis*-$[Pt^{II}(MNZ)(OH)(H_2O)_2]^+$ and (b) *trans*-$[Pt^{II}(MNZ)_2(H_2O)(N_3)]^+$. Relative Gibbs free energies (kcal mol^{-1}) are calculated with respect the initial adducts whose energy was set equal to zero. The optimized structures of the intercepted transition states are reported above the arrows. For the sake of clarity, in depicting the transition state structures, the metronidazole (MNZ) ligand is replaced by a yellow sphere.

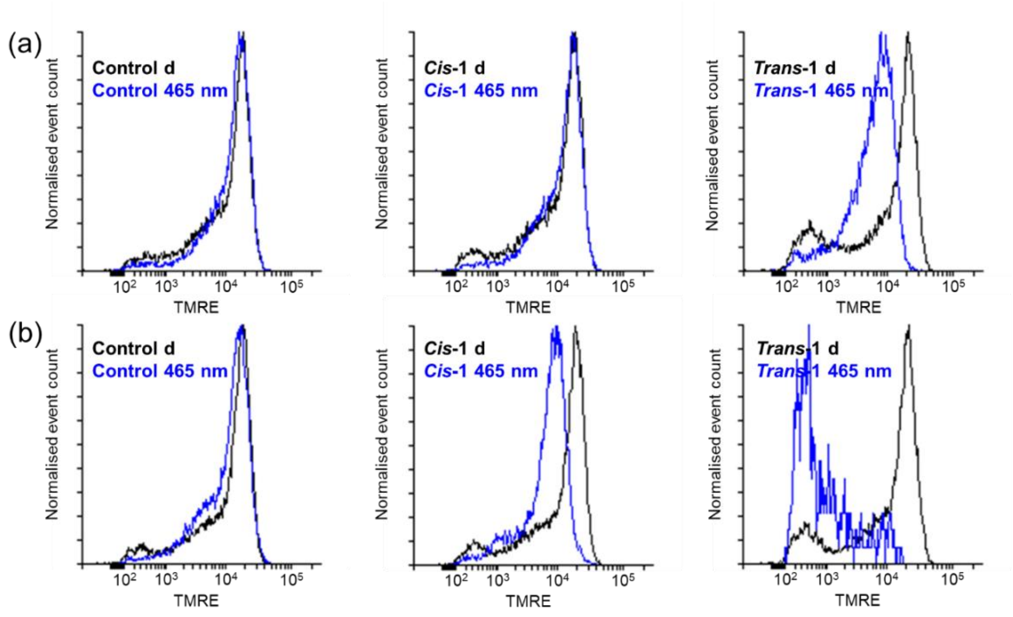

Figure S15. Mitochondrial membrane potentials analysed by flow cytometry for SW780 cells under normoxia treated with *Cis*-**1** and *Trans*-**1** (a for 10 µM and b for 20 µM) in the dark (2 h) or 1 h incubation and 1 h irradiation (465 nm), and 72 h further incubation, then stained by TMRE ($\lambda_{ex}/\lambda_{em}$ = 561/570–600 nm).

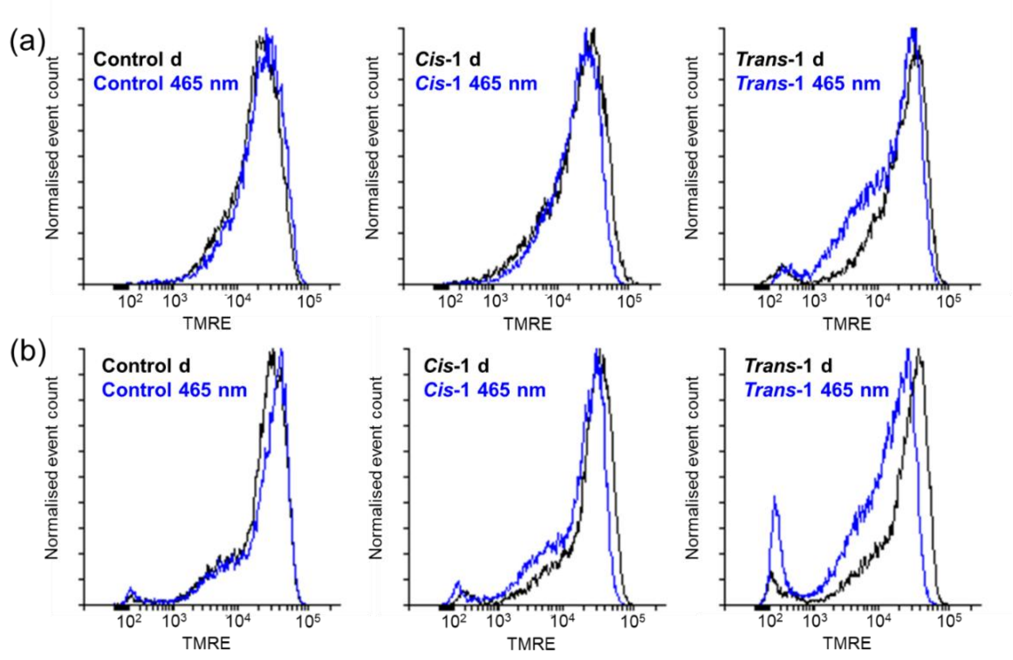

Figure S16. Mitochondrial membrane potential analysed by flow cytometry for SW780 cells under hypoxia treated with (a for 10 µM and b for 20 µM) *Cis*-**1** and *Trans*-**1** in the dark (2 h) or 1 h incubation and 1 h irradiation (465 nm), and 72 h further incubation, then stained by TMRE ($\lambda_{ex}/\lambda_{em}$ = 561/570–600 nm).

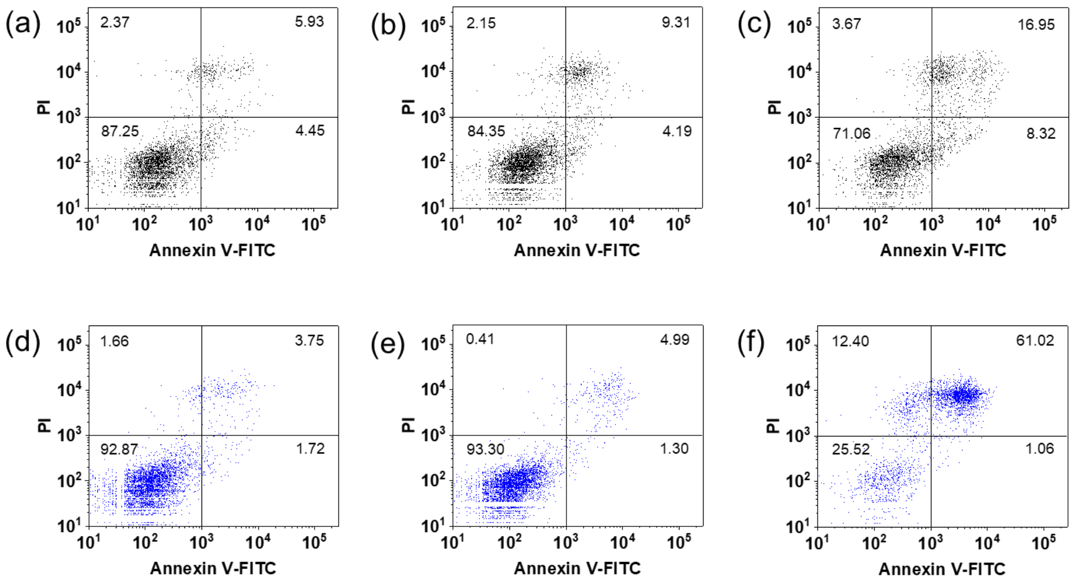

Figure S17. Cell apoptosis assays for SW780 cells under normoxia double-stained by Annexin V-FITC/PI ($\lambda_{ex}/\lambda_{em}$ = 488/500–560 nm for Annexin V-FITC, $\lambda_{ex}/\lambda_{em}$ = 488/645–735 nm for PI) and analyzed by flow cytometry. a) untreated SW780 cells in the dark and d) irradiated with blue light (465 nm); b) SW780 cells treated with *Cis*-**1** (20 µM) in the dark and e) irradiated with blue light; c) SW780 cells treated with *Trans*-**1** (20 µM) in the dark and f) irradiated with blue light.

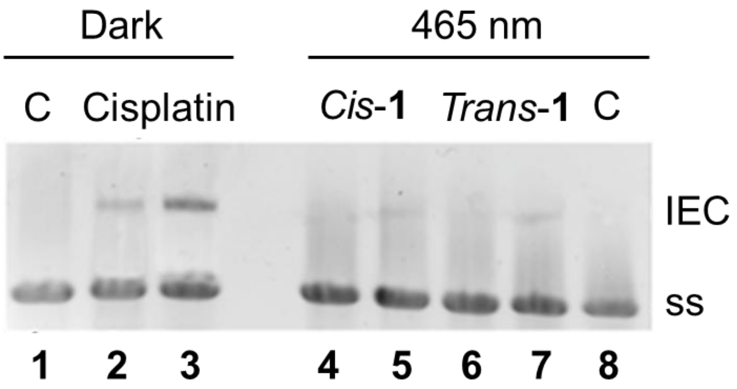

Figure S18. Autoradiograms of denaturing 1% agarose gel of linearized pDNA; the interstrand cross-linked DNA appears as the top bands (IECs) migrating on the gel more slowly than the single-stranded (ss) DNA (contained in the bottom bands). Plasmid linearized by EcoRI was incubated for 24 h with cisplatin, *Cis*-**1**, and *Trans*-**1** at r_b values of 0 (control, lanes 1 and 8), 0.0005 (lanes 2, 4, 6) or 0.001 (lanes 3,5,7) (r_b= number of Pt bound per nucleotide residue).

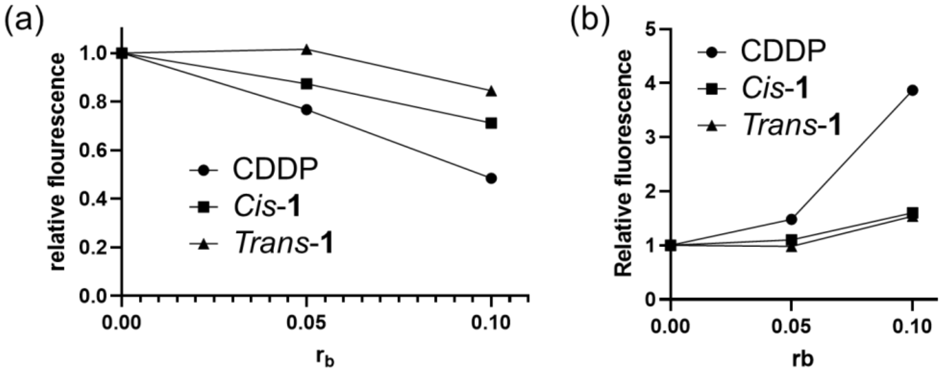

Figure S19. (a) Plots of EtBr fluorescence versus r_b for DNA modified by Pt-complexes for 24h (1 h irrad 465 nm + 23 h in the dark for *Cis*-**1** and *Trans*-**1**, samples with cisplatin were incubated in the dark for 24 h). (b) Change in the relative fluorescence of Tb^{3+} ions bound to double-helical *ct*DNA modified by platinum complexes. The fluorescence of the untreated DNA-EtBr was arbitrarily set at 1.

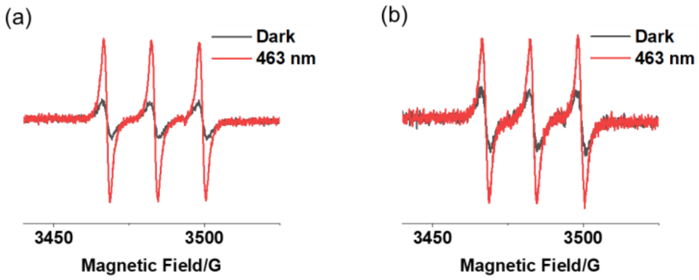

Figure S20. EPR spectra for complexes (2.5 mM) (a) *Cis*-**1** and (b) *Trans*-**1** in the presence of TEMP (20 mM) in acetonitrile (containing 5% DMSO) to trap singlet oxygen; Dark (—); blue light (—, 463 nm, 20 min). The experimental trace is for accumulation of 5 scans (conversion time 10.24 ms, time constant 10.24 ms, and sweep time 20.97 s for each scan).

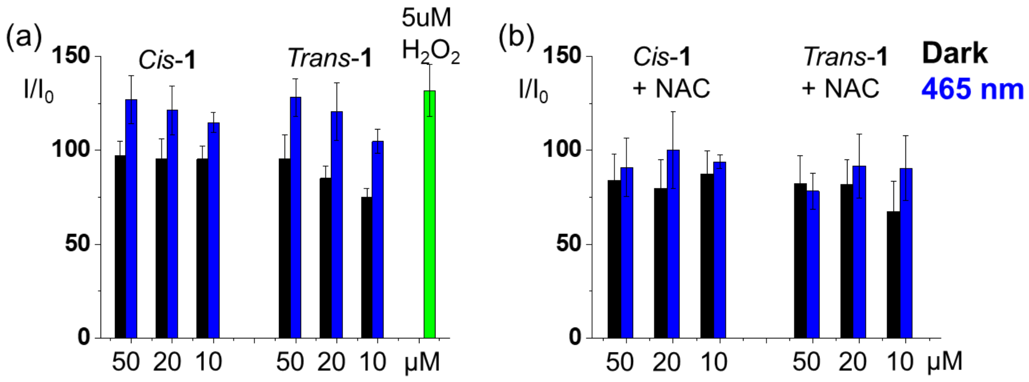

Figure S21. Relative fluorescence intensity of SW780 cells treated with *Cis*-**1** or *Trans*-**1** (2 h or 1 h in the dark and 1 h irradiation, 465 nm) in the (a) absence and (b) presence of 10 mM antioxidant *N*-acetyl-L-cysteine (NAC), then probed with DCFH-DA (20 µM, λ_{ex} = 485 nm, λ_{em} = 521–539 nm). H_2O_2 (5 µM) was used as a positive control.

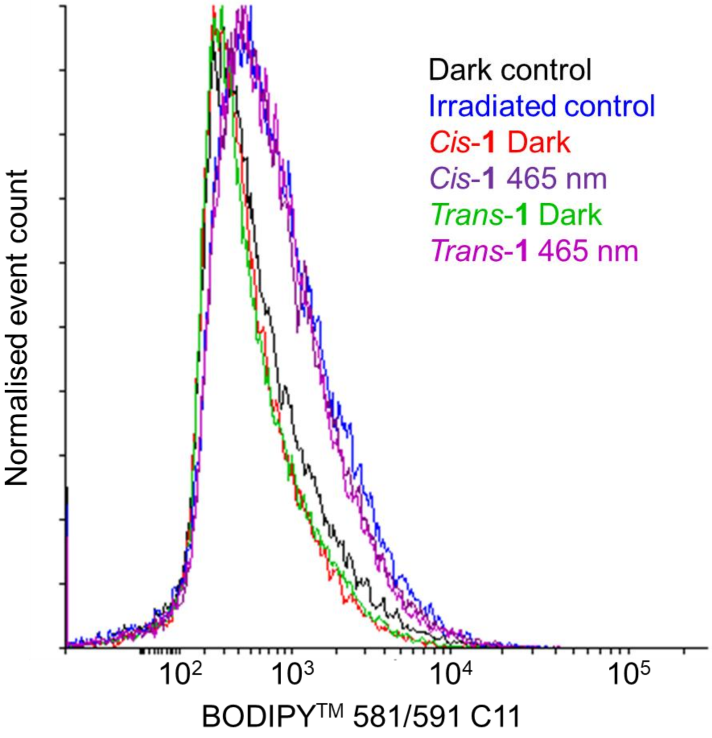

Figure S22. Lipid peroxidation assay for SW780 cells stained by BODIPY^{TM} 581/591 C11 ($\lambda_{ex}/\lambda_{em}$ = 488/500–560 nm) and analyzed by flow cytometry. Complex concentration is 20 µM. X-axis indicates the intensity of emission from oxidized BODIPY^{TM} 581/591 C11.

References

1. Dolomanov, O. V.; Bourhis, L. J.; Gildea, R. J.; Howard, J. A. K.; Puschmann, H.; *J. Appl. Crystallogr*. **2009**, *42*, 339−341.
2. Sheldrick, G. M. *Acta Crystallogr*., Sect. A: Found. Adv. **2015**, *71*, 3−8.
3. Sheldrick, G. M. *Acta Crystallogr., Sect. C: Struct. Chem*. **2015**, *71*, 3−8.
4. Vichai, V.; Kirtikara, K. *Nat. Protoc*. **2006**, *1*, 1112−1116.
5. Butour, J. L.; Alvinerie, P.; Souchard, J. P.; Colson, P.; Houssier, C.; Johnson, N. P. *Eur. J. Biochem*. **1991**, *202*, 975–980
6. Balcarova, Z.; Brabec, V. *Biophys. Chem*. **1989**, *33* (1), 55−61.
7. Gaussian 16, Revision B.01, Frisch, M. J.; Trucks, G. W.; Schlegel, H. B.; Scuseria, G. E.; Robb, M. A.; Cheeseman, J. R.; Scalmani, G.; Barone, V.; Petersson, G. A.; Nakatsuji, H.; Li, X.; Caricato, M.; Marenich, A. V.; Bloino, J.; Janesko, B. G.; Gomperts, R.; Mennucci, B.; Hratchian, H. P.; Ortiz, J. V.; Izmaylov, A. F.; Sonnenberg, J. L.; Williams-Young, D.; Ding, F.; Lipparini, F.; Egidi, F.; Goings, J.; Peng, B.; Petrone, A.; Henderson, T.; Ranasinghe, D.; Zakrzewski, V. G.; Gao, J.; Rega, N.; Zheng, G.; Liang, W.; Hada, M.; Ehara, M.; Toyota, K.; Fukuda, R.; Hasegawa, J.; Ishida, M.; Nakajima, T.; Honda, Y.; Kitao, O.; Nakai, H; Vreven, T.; Throssell, K.; Montgomery Jr., J. A.; Peralta, J. E.; Ogliaro, F.; Bearpark, M. J.; Heyd, J. J.; Brothers, E. N.; Kudin, K. N.; Staroverov, V. N.; Keith, T. A.; Kobayashi, R.; Normand, J.; Raghavachari, K.; Rendell, A. P.; Burant, J. C.; Iyengar, S. S.; Tomasi, J.; Cossi, M.; Millam, J. M.; Klene, M.; Adamo, C.; Cammi, R.; Ochterski, J. W.; Martin, R. L.; Morokuma, K.; Farkas, O.; Foresman, J. B.; Fox, D. J. *GaussView 5.0. Wallingford, E.U.A.* **2016**.
8. Lee, C.; Yang, W.; Parr, R. G. *Phys. Rev. B* **1988**, *37* (2), 785–789.
9. Grimme, S.; Antony, J.; Ehrlich, S.; Krieg, H. *J. Chem. Phys*. **2010**, *132* (15), 154104.
10. Becke, A. D. *J. Chem. Phys*. **1993**, *98* (7), 5648–5652.
11. Andrae, D.; Häußermann, U.; Dolg, M.; Stoll, H.; Preuß, H. *Theoret. Chim. Acta* **1990**, *77* (2), 123–141.
12. Fukui, K. *J. Phys. Chem*. **1970**, *74* (23), 4161–4163.
13. Perdew, J. P.; Chevary, J. A.; Vosko, S. H.; Jackson, K. A.; Pederson, M. R.; Singh, D. J.; Fiolhais, C. *Phys. Rev. B* **1992**, *46* (11), 6671–6687.
14. Perdew, J. P.; Wang, Y. *Phys. Rev. B* **1992**, *45* (23), 13244–13249.
15. Yanai, T.; Tew, D. P.; Handy, N. C. *Chem. Phys. Lett*. **2004**, *393* (1), 51–57.
16. Ernzerhof, M.; Scuseria, G. E. *J. Chem. Phys*. **1999**, *110* (11), 5029–5036.
17. Adamo, C.; Barone, V. *J. Chem. Phys*. **1999**, *110* (13), 6158–6170.
18. Zhao, Y.; Schultz, N. E.; Truhlar, D. G. *J. Chem. Phys*. 2005, *123* (16), 161103.

19. Zhao, Y.; Truhlar, D. G. *Theor. Chem. Acc.* **2008**, *120* (1), 215–241.
20. Zhao, Y.; Truhlar, D. G. *J. Chem. Phys*. **2006**, *125* (19), 194101.
21. Peverati, R.; Truhlar, D. G. *J. Phys. Chem. Lett*. **2011**, *2* (21), 2810–2817.
22. Plasser, F. *J. Chem. Phys.* **2020**, *152* (8), 084108.
23. Neese, F. *Comp. Mol. Sci.* **2012**, *2* (1), 73–78.
24. Neese, F. *Comp.l Mol. Sci.* **2018**, *8* (1), e1327.
25. Escudero, D.; Heuser, E.; Meier, R. J.; Schäferling, M.; Thiel, W.; Holder, E. *Chem. Eur. J*. **2013**, *19* (46), 15639–15644.
26. Escudero, D.; González, L. *J. Chem. Theory Comput*. **2012**, *8* (1), 203–213.
27. Mackay, F. S.; Woods, J. A.; Heringova, P.; Kašpárková, J.; Pizarro, A. M.; Moggach, S. A.; Parsons, S.; Brabec, V.; Sadler, P. J. *Proc. Natl. Acad. Sci. U. S. A.* **2007**, *104*, 20743–20748.
28. Shi, H.; Ward-Deitrich, C.; Ponte, F.; Sicilia, E.; Goenaga-Infante, H.; Sadler, P. J. *Dalton Trans.* **2024**, *53* (31), 13044–13054.
29. Shi, H.; Ponte, F.; Grewal, J. S.; Clarkson, G. J.; Imberti, C.; Hands-Portman, I.; Dallmann, R.; Sicilia, E.; Sadler, P. J. *Chem. Sci.* **2024**, *15* (11), 4121–4134.
30. H. Shi, G. J. Clarkson, P. J. Sadler, *Inorg. Chem. Front*., **2024**, *11* (22), 7898–7909.
